# ATM Modulates the Loading of Recombination Proteins onto a Chromosomal Translocation Breakpoint Hotspot

**DOI:** 10.1371/journal.pone.0013554

**Published:** 2010-10-27

**Authors:** Jiying Sun, Yukako Oma, Masahiko Harata, Kazuteru Kono, Hiroki Shima, Aiko Kinomura, Tsuyoshi Ikura, Hidekazu Suzuki, Shuki Mizutani, Roland Kanaar, Satoshi Tashiro

**Affiliations:** 1 Department of Cellular Biology, RIRBM, Hiroshima University, Hiroshima, Japan; 2 Laboratory of Molecular Biology, Graduate School of Agricultural Science, Tohoku University, Sendai, Japan; 3 Laboratory of Chromatin Regulatory Network, RBC, Kyoto University, Kyoto, Japan; 4 Department of Pediatrics and Developmental Biology, Graduate Medical School, Tokyo Medical and Dental University, Tokyo, Japan; 5 Department of Cell Biology and Genetics, Cancer Genomics Center, Department of Radiation Oncology, Erasmus MC, Rotterdam, The Netherlands; National Institute on Aging, United States of America

## Abstract

Chromosome translocations induced by DNA damaging agents, such as ionizing radiation and certain chemotherapies, alter genetic information resulting in malignant transformation. Abrogation or loss of the ataxia-telangiectasia mutated (ATM) protein, a DNA damage signaling regulator, increases the incidence of chromosome translocations. However, how ATM protects cells from chromosome translocations is still unclear. Chromosome translocations involving the MLL gene on 11q23 are the most frequent chromosome abnormalities in secondary leukemias associated with chemotherapy employing etoposide, a topoisomerase II poison. Here we show that ATM deficiency results in the excessive binding of the DNA recombination protein RAD51 at the translocation breakpoint hotspot of 11q23 chromosome translocation after etoposide exposure. Binding of Replication protein A (RPA) and the chromatin remodeler INO80, which facilitate RAD51 loading on damaged DNA, to the hotspot were also increased by ATM deficiency. Thus, in addition to activating DNA damage signaling, ATM may avert chromosome translocations by preventing excessive loading of recombinational repair proteins onto translocation breakpoint hotspots.

## Introduction

Recurring chromosome translocations are often associated with specific types of leukemia/cancer and DNA damaging agents[1]. Breakpoints of these chromosome translocations have been shown to cluster within restricted regions in or around the genes implicated in the translocations. Chromosome translocations involving 11q23 are one of the most common chromosome abnormalities observed in secondary and infantile leukemias [2], [3]. Among drugs used for anti-cancer chemotherapy, etoposide, a topoisomerase II inhibitor, has been clearly associated with the therapy-related leukemia carrying 11q23 chromosome translocations [4], [5]. Most chromosomal translocation breakpoints in 11q23 are located within an 8.3-kb breakpoint cluster region (BCR) spanning from exon 7 to 13 of the MLL gene [6], [7]. However, how etoposide induces 11q23 chromosome translocations in this region is largely unknown [8].

DNA damage leads to activation of DNA damage response and repair pathways. In normal cells, the ataxia-telangiectasia mutated (ATM) protein regulates the DNA damage response in reaction to DNA double-strand breaks (DSBs) through its kinase activity [9]. Altered function of ATM plays pathologic roles in the development of leukemia/lymphoma and cancer including leukemia with MLL translocations [10], [11]. Moreover, an increase of 11q23 translocations is observed in an ATM kinase activity deficient fibroblast cell line AT5BIVA [12]. Although these findings indicate the involvement of ATM in chromosome translocations involving 11q23, how ATM deficiency renders the BCR in the MLL gene highly recombinogenic after etoposide treatment is still unclear.

Homologous recombination (HR) is a versatile DNA repair mechanism because it can promote the repair of a variety of lesions including DSBs, single-strand gaps and stalled DNA replication forks. RAD51 is one of the key proteins for DNA repair by HR because it mediates homologous pairing and strand exchange between DNA duplexes [13]. Interestingly, the elevated RAD51 expression levels in tumor cells have been suggested to contribute to genomic instability by stimulating aberrant recombination between short repetitive elements and homologous sequences [14], [15], [16]. Moreover, increased RAD51 expression by introducing a RAD51 expression vector in a mouse embryonic stem cell line promotes aneuploidy and chromosomal rearrangement [17]. These findings suggest a link between increased levels of RAD51 and chromosomal instability.

Here, we identified the BCR as the first native human chromosomal DNA locus where RAD51, Replication protein A (RPA) and INO80, a recombinational repair associated chromatin remodeler [18], accumulate upon etoposide treatment. Importantly, ATM deficiency enhanced the etoposide-induced accumulation of RAD51, RPA, and INO80 at the BCR. Thus, in addition to activating DNA damage signaling, ATM modulates the loading of recombinational repair proteins onto translocation breakpoint hotspots to avoid inappropriate recombination leading to chromosome translocation.

## Results

### ATM and RAD51 are involved in 11q23 chromosomal translocations

**Figure 1:**
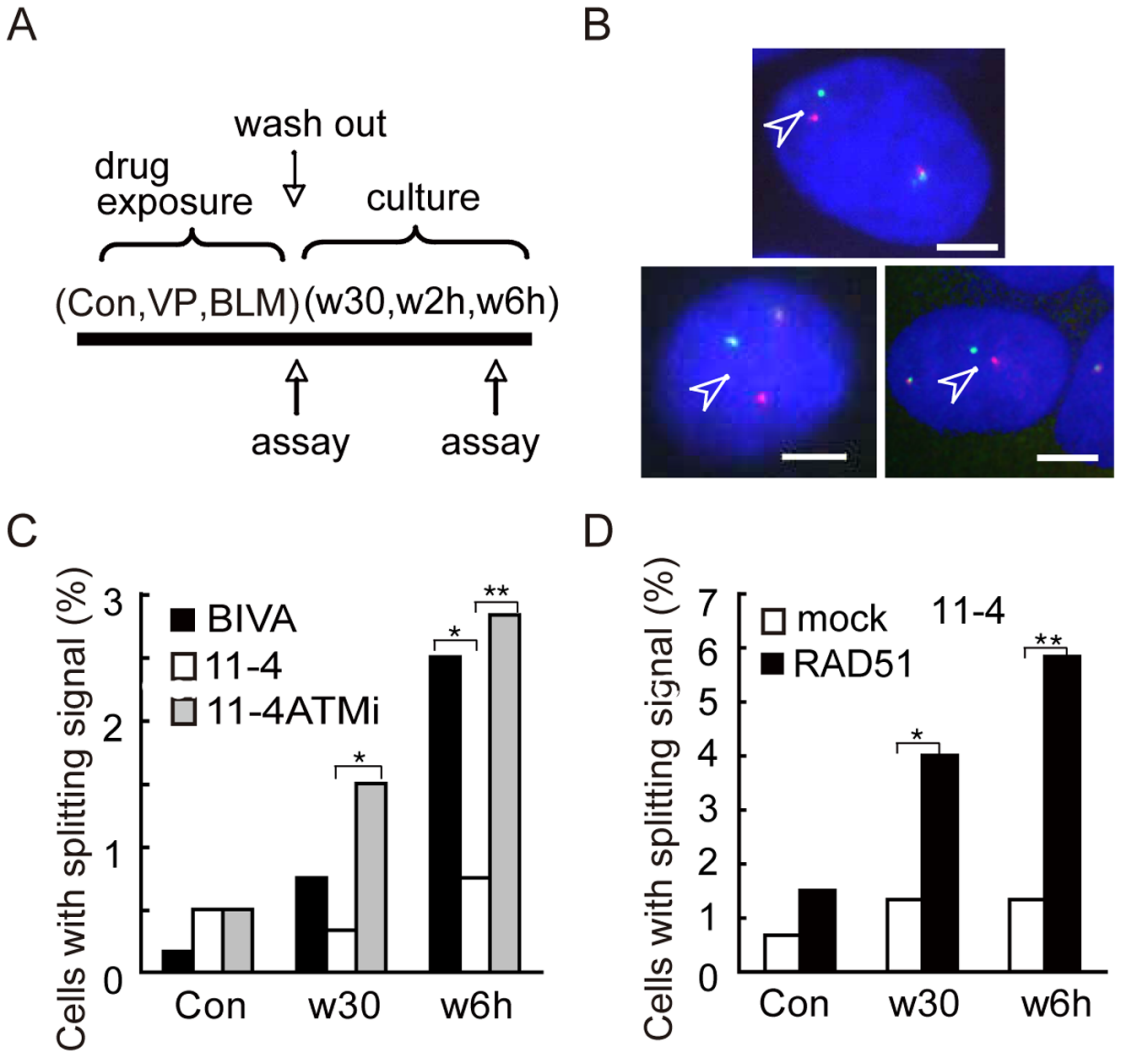
MLL rearrangement after etoposide treatment. (**A**) Schematic diagram of etoposide (VP) or Bleomycin (BLM) exposure and subsequent culture for recovery. (**B-D**) Dual-color FISH analysis of AT5BIVA and 11-4 cells, cultured for 30 min (w30) and 6 hours (w6h) after etoposide exposure (Con; unexposed cells). The cells were hybridized with paired probes spanning the MLL gene with overlap in the BCR (centromeric side in green, telomeric side in red). (**B**) The representative images of FISH (BIVA w6h) are shown. Arrowheads indicate the split signals (separated >1 µm). Scale bar: 5 µm. (**C**) The percentage of cells with split signals was significantly increased for AT5BIVA cells or the ATM kinase inhibitor treated 11-4 cells compared to normal 11-4 cells cultured for 30 min (w30) or 6 hours (w6h) in normal medium after etoposide treatment (*P<0.05, **P<0.0001 as determined by the Z test of homogeneity for independent samples, respectively). (**D**) 11-4 cells were transfected with a RAD51 expression vector (RAD51) or an empty vector (mock). Twenty two hours after transfection, cells were treated with etoposide and cultured for 30 min (w30) and 6 hours (w6h) in normal medium (Con; unexposed cells). The percentage of cells with split signals was significantly higher in RAD51 overexpressing 11-4 cells compared to empty vector transfected cells after culturing for 30 min and 6 hours in normal medium (*P<0.01, **P<0.0001 as determined by the Z test).

To examine the involvement of ATM kinase and recombination proteins in 11q23 chromosomal translocations, we first analyzed the rearrangement of the MLL gene after etoposide treatment in ATM-deficient AT5BIVA cells and a clone of AT5BIVA complemented with chromosome 11 (11-4), which carries the *ATM* gene (Figure 1A). FISH analysis was performed using the 2-color paired FISH probes located on either side of the MLL gene. Since the paired probes span a genomic region of ∼600 kb and have little overlap, the MLL gene was most often detected as side-by-side red and green signals of about 0.2 µm in diameter (Figure S1). Therefore, when the centers of the red and green signals were separated by>1 µm, we refer to their arrangement as a ‘split signals’, which is indicative of a rearranged MLL gene (Figure 1B). A significant increase in cells carrying split MLL gene signals at 6, 36 and 48 hours after etoposide exposure was observed when the cells were ATM deficient and not when they were ATM proficient (Figures 1B, C and S2). Moreover, treatment of 11-4 cells with KU55933, a specific inhibitor of ATM kinase, increased the incidence of 11-4 cells with the split signals after etoposide treatment to the level comparable to that of BIVA cells (Figure 1C). Apoptosis can potentially induce DNA breaks detected as a split signals. However, there were actually less apoptotic cells in the treated AT5BIVA cell population than in the 11-4 cell at 6 hours after etoposide treatment (Figure S3). Furthermore, it is unlikely that the split signals are due to DSBs ends that have become untethered due to possible reduced function of Ku in AT5BIVA cells compared to 11-4 cells, because immunoblotting and gel-shift analyses showed no significant difference in the expression and DNA binding activity of Ku80 between the cells (Figure S4). Moreover, long distance inverse PCR analysis identified more clones containing chromosome translocation breakpoints within the BCR in AT5BIVA cells than in 11-4 cells (Figures S5). Therefore, consistent with previous reports, these findings support the notion that ATM deficiency increases 11q23 chromosome translocations after etoposide exposure [12].

Since RAD51 overexpression has been implicated in chromosome translocations in general [17], we investigated whether increased RAD51 expression specifically promotes 11q23 chromosome translocations. RAD51 expression levels were transiently elevated by transfection of 11-4 cells with a human RAD51 cDNA expression vector. We estimated the transfection efficiency at approximately 44% as determined by immunofluorescence analysis (Figure S6). Immunoblotting of whole-cell extracts indicated an approximately 7-fold increase in the level of RAD51 protein compared to extract from non-transfected cells. We then analyzed the incidence of 11q23 chromosomal translocations after etoposide treatment in 11-4 cells with increased RAD51 expression. The percentage of cells carrying split signals in the dual-color FISH analysis was significantly increased in 11-4 cells overexpressing RAD51 after etoposide treatment (Figure 1D). This finding suggests that increased RAD51 expression facilitates 11q23 chromosomal translocations after etoposide treatment. In contrast to ATM deficiency, RAD51 overexpression substantially increases the amount of RAD51 in chromatin fraction (Figure S7). Therefore, the mechanisms for the effects of RAD51 overexpression and ATM deficiency on 11q23 chromosome translocations could be different.

### Loading of RAD51 onto the BCR after etoposide treatment is modulated by ATM

**Figure 2:**
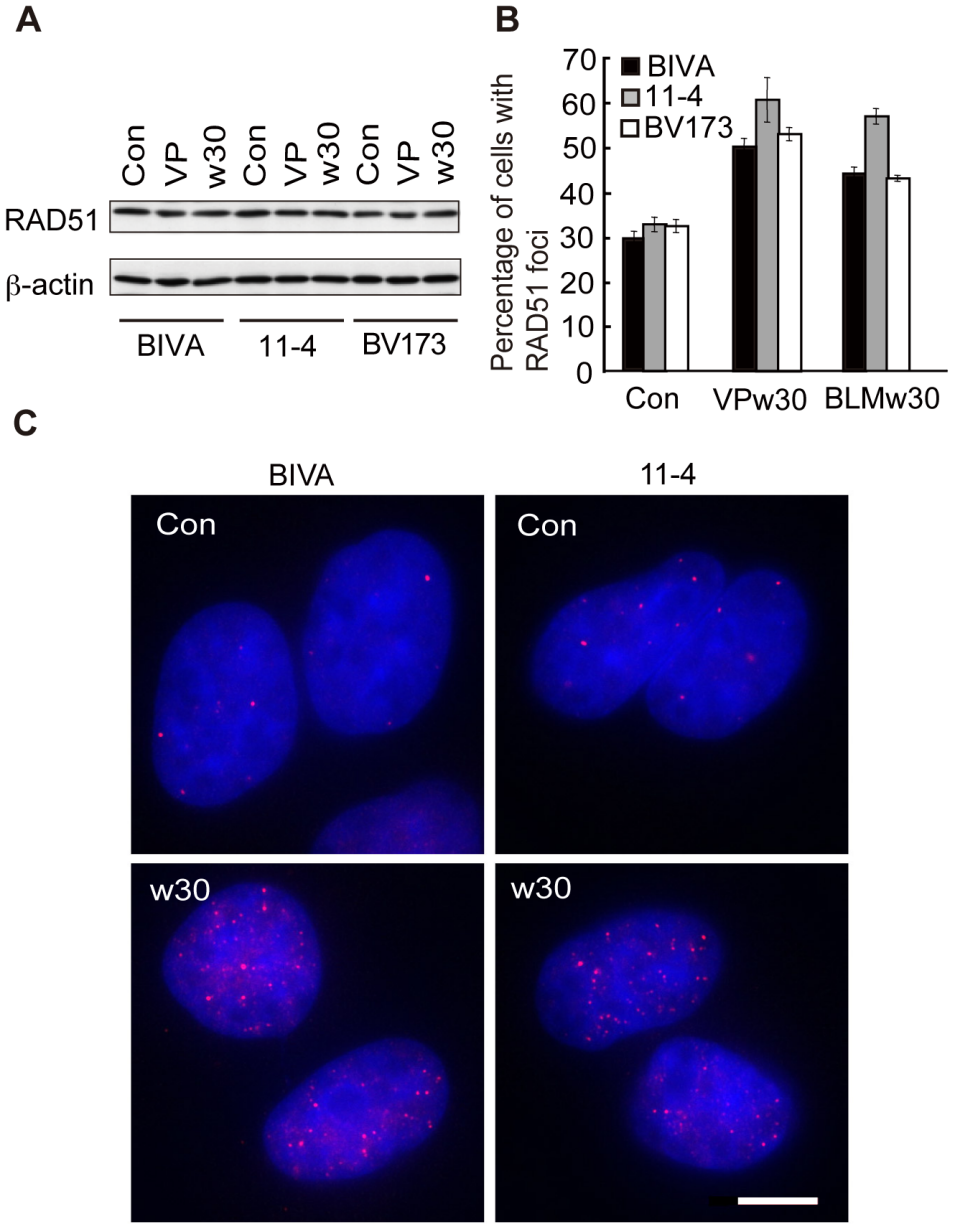
Focus formation and expression of RAD51 in ATM deficient cells. (**A**) Immunoblotting analysis of BV173, AT5BIVA and 11-4 cells using an anti-RAD51 antibody. Cells were treated with etoposide (w30) or DMSO (Con) for 10 min and cultured for 30 min in normal medium. β-actin was used as a loading control. (**B**) The percentage of RAD51 foci positive AT5BIVA, 11-4 and BV173 cells was significantly increased after etoposide or bleomycin treatment (p<0.001, as determined by the Z test of homogeneity for independent samples). Values represent the means ± SE from three to four independent experiments (n>200 cells for each). (**C**) Focus formation of RAD51 in AT5BIVA and 11-4 cells after etoposide exposure. RAD51 nuclear foci were detected by immunofluorescence staining using anti-RAD51 antibodies. Scale bar: 5 µm.

**Figure 3:**
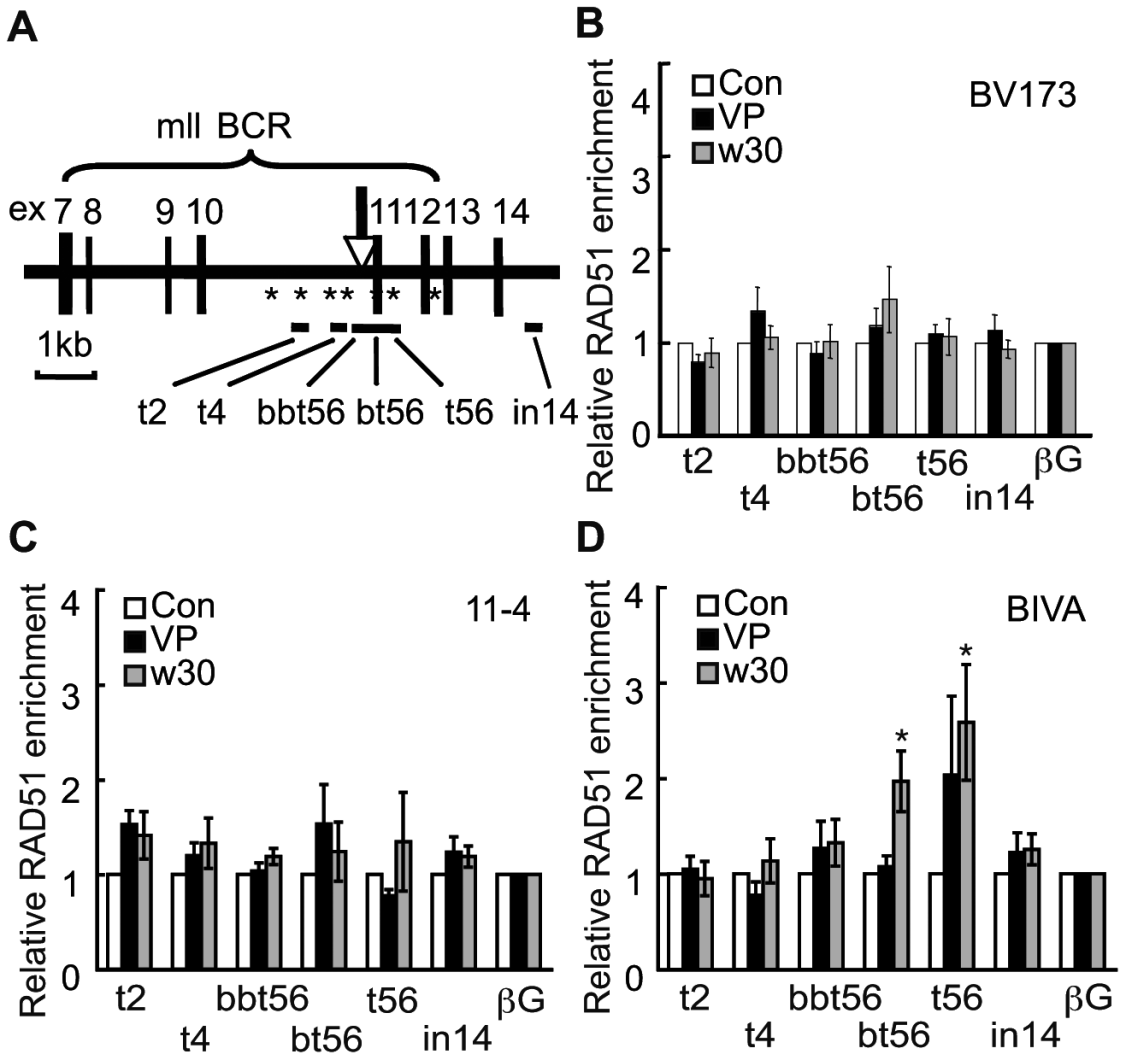
Binding of RAD51 to breakpoint clustering region in the MLL gene after etoposide treatment. (**A**) Schematic representation of the BCR in the MLL gene. The arrow indicates the translocation breakpoint hotspot identified in treatment-related leukemias. Asterisks represent topoisomerase II consensus sites. Positions of primer sets used in real-time PCR analysis (t2, t4, bbt56, bt56, t56 and in14) are indicated. (**B-D**) ChIP analysis of BV173 (B), 11-4 (C) and AT5BIVA (D) cells was performed with anti-RAD51 antibodies, immediately after etoposide (VP), or vehicle (Con) exposure, and 30 min after recovery in normal medium (w30). DNA was analyzed by real-time PCR using the primers indicated in (A) and that for a control region in the β-globin gene (βG). Values represent the means ± SE from five independent experiments. *: P<0.05 (compared to βG by Student's t test).

Since increased levels of RAD51 promoted 11q23 chromosomal translocations after etoposide treatment, we examined whether etoposide treatment and/or ATM kinase activity affect RAD51 expression. Immunoblotting analysis of human lymphoid cell line BV173, AT5BIVA and 11-4 cells showed that RAD51 expression was not induced by etoposide treatment or ATM deficiency (Figure 2A). RAD51 is also known to form nuclear foci after induction of DNA damage by ionizing irradiation or certain chemicals [19], [20]. Immunofluorescence staining showed that after etoposide treatment RAD51 foci positive cells increased to similar levels in AT5BIVA, 11-4 and BV173 cells (Figures 2B and C). These findings led us to speculate that RAD51 may be specifically loaded on the BCR after etoposide exposure resulting in illegitimate recombination leading to 11q23 translocation. If so, RAD51 loading is predicted to be enhanced in the absence of ATM activity because ATM-deficient cells display an increased translocation frequency. To test this hypothesis, we examined the recruitment of RAD51 to the BCR in AT5BIVA cells using chromatin immunoprecipitation (ChIP) followed by real-time polymerase chain reaction (PCR). We designed 6 primer sets (t2, t4, bbt56, bt56, t56, in14) to analyze RAD51 binding in a region around the approximately 20 base pairs (bps) translocation breakpoint hotspot (Figure 3A and Figure S8) [4], [5]. First, we tested the amplification efficiency of DNA extracted from cells after etoposide treatment by PCR. Real-time PCR analysis using these 6 primer sets showed no significant differences in the amplification efficiency of DNA extracted from cells before and after etoposide treatment (Figure S9). ChIP analysis of BV173 and 11-4 cells using anti-RAD51 antibodies provided a hint that upon etoposide treatment RAD51 accumulated to slightly higher levels in the contiguous bt56 and t56 regions within the BCR compared to adjacent regions and a control region in the β-globin gene locus on chromosome 11p15 (Figures 3B and C). Interestingly, ChIP analysis of AT5BIVA revealed that 30 min after recovery from etoposide treatment RAD51 protein accumulated to significantly higher levels in the bt56 and t56 regions within the BCR compared to the closely located t2, t4 and in14 regions as well as the β-globin control region (Figure 3D). Higher levels of the RAD51 binding to the BCR in AT5BIVA cells could be observed after treatment of cells with an increased dose of etoposide (Figure S10). As a control we used bleomycin, a DNA damaging agent that is not associated with 11q23 chromosome translocation. Like etoposide, bleomycin induced RAD51 focus formation and did not affect the expression of RAD51 (Figure 2B and Figure S11A). However, compared with etoposide exposure, bleomycin treatment did not result in a significant increase of RAD51 in the BCR in 11-4 and AT5BIVA cells (Figures S11B and C).

Finally, treatment of 11-4 cells with the ATM kinase inhibitor increased the binding of RAD51 to the BCR after etoposide treatment (Figure S12). These findings indicate that kinase activity of ATM is required for the proper regulation of the RAD51 binding to the BCR after etoposide treatment. Since ATM deficiency and overexpression of RAD51 could enhance the effect of etoposide to induce 11q23 chromosomal translocations, aberrant binding of RAD51 to the BCR by ATM deficiency may be responsible for the 11q23 chromosome translocations.

### Binding of RPA to the BCR after etoposide treatment in ATM deficient cells

**Figure 4:**
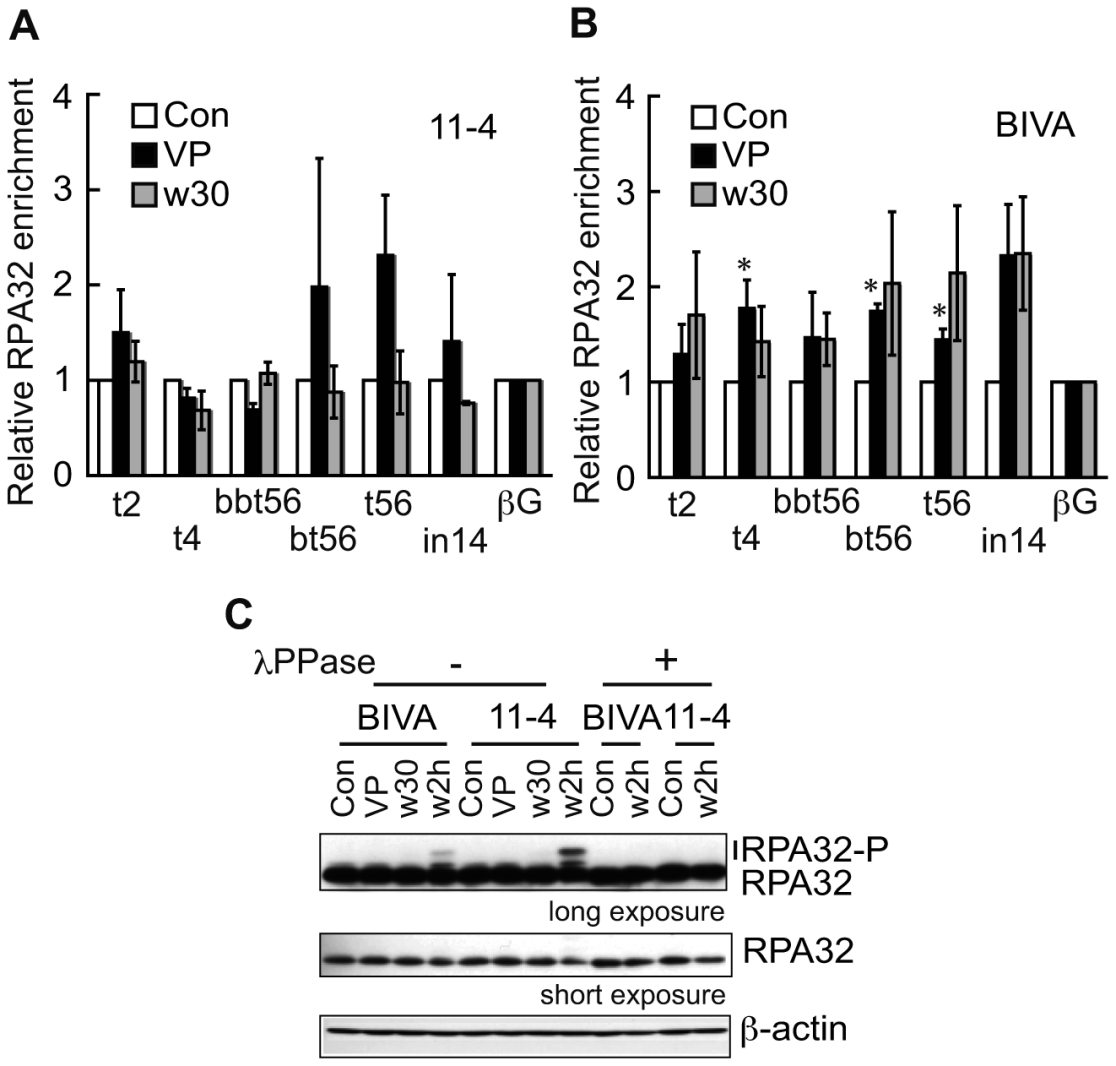
RPA is loaded on the BCR after etoposide treatment. (**A, B**) ChIP analysis after etoposide treatment of 11-4 (A) and AT5BIVA (B) cells using antibodies against RPA was performed as described in Figure 3. Values represent the means ± SE from three independent experiments. *P<0.05 (compare to βG by Student's t test). (**C**) Immunoblotting analysis of AT5BIVA and 11-4 cells using anti-RPA32 antibodies. Cells were treated with etoposide for 10 min (VP) and allowed to recover in normal medium for 30 min (w30) or 2 hours (w2h). Slower migrating bands, indicated as RPA32-P, in the long exposed blot were lost by λ-phosphatase (λPPase) treatment. β-actin was used as a loading control.

Single-stranded DNA is the recombinogenic form of DNA onto which RAD51 forms its active nucleoprotein filament that is capable of engaging the partner DNA during HR. Once single-stranded DNA is formed in cells in response to DNA damage, and before formation of the RAD51 nucleoprotein filament, it is first coated with replication protein A (RPA) [21], [22]. Therefore, we examined whether the binding of RPA to the BCR after etoposide treatment differed in ATM-proficient versus ATM-deficient cells. Interestingly, ChIP analysis of 11-4 cells, immediately after etoposide removal, using anti-RPA antibodies revealed etoposide-induced enrichment of RPA in the BCR (Figure 4A). Since the anti-RPA antibodies immunoprecipitates both phosphorylated and non-phosphorylated forms of RPA (Figure S13), this result indicates that RPA accumulates in the BCR as a consequence of etoposide treatment. The ATM proficient 11-4 cells did recover from the etoposide-induced insult because 30 min after recovery from the exposure RPA levels returned to normal (Figure 4A). The ATM deficient AT5BIVA cells also displayed an increase of RPA bound to the BCR compared to the control region, but with two noticeable differences (Figure 4B). First, RPA accumulation was more extensive since it was observed in regions adjacent to bt56/t56. Second, at 30 min after removal of the etoposide local RPA levels remained high. Since the expression level of RPA was not influenced by etoposide treatment or ATM kinase activity (Figure 4C), ATM may control the appropriate loading and release of RPA on damaged chromatin in the BCR after etoposide treatment. These findings support the notion that ATM represses the aberrant overloading of RPA, together with RAD51, on the BCR after etoposide treatment by possibly promoting the recovery of the etoposide-induced insult to DNA.

### ATM regulates the binding of INO80 to the BCR

**Figure 5:**
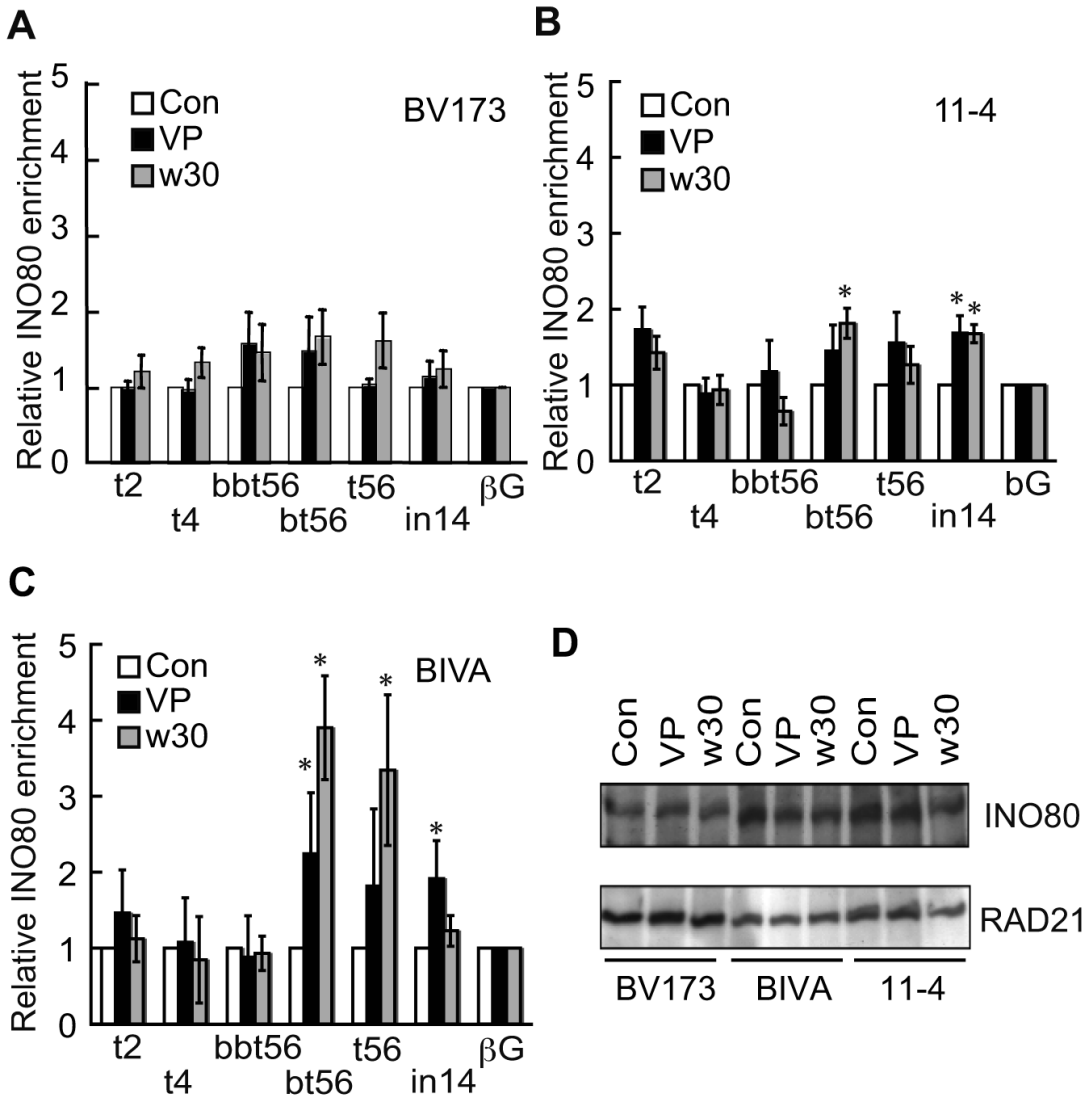
INO80 loading on the BCR after etoposide treatment. (**A-C**) ChIP analysis of BV173 (A), 11-4 (B) and AT5BIVA (C) cells after etoposide exposure was performed with antibodies against INO80 as described in Figure 3. Values are the means ± SE from three independent experiments. (**D**) Immunoblotting analysis of BV173, AT5BIVA and 11-4 cells was performed using anti-INO80 antibodies. RAD21 was used as a loading control. * P<0.05 (compared to βG by Student's t test).

The INO80 chromatin remodeling complex has been implicated in the DNA damage response by facilitating single-stranded DNA formation, a critical step in HR, and the displacement of RPA with RAD51 in yeast cells [22], [23], [24]. Recently, INO80 has been shown to play a role in mammalian HR [18]. Based on these findings, we investigated the recruitment of INO80 to the BCR by ChIP. Consistent with the notion that etoposide induces an aberrant DNA structure in the BCR, resulting in a different chromatin configuration as marked by increased RPA and RAD51 levels, the level of INO80 also increased in the BCR compared to the control locus upon etoposide exposure (Figures 5A and B). Importantly, in the absence of ATM activity there was a more significant etoposide-induced enrichment of INO80 at the BCR (Figure 5C). Since the expression of INO80 was regulated independently of ATM function and etoposide exposure (Figure 5D), our findings suggest that ATM is required to properly regulate RAD51-mediated DNA repair of etoposide-induced DNA damage to which the BCR in the MLL locus is particularly susceptible and that in its absence progression of repair stalls resulting in excessive accumulation of INO80 and RAD51 on the BCR.

## Discussion

In this study, we show that in the absence of ATM activity the chromatin remodeler INO80, as well as the HR repair proteins RPA and RAD51 accumulate at the BCR after etoposide treatment. Since RAD51 makes DNA highly recombinogenic [17], [25], the aberrant loading of RAD51 on the BCR could increase the recombinogenic activity of damaged chromatin within the region. This may interfere with the normal repair process resulting in the increased incidence of 11q23 chromosome translocation in ATM kinase deficient cells.

ATM deficiencies increased the incidence of cells with the split MLL gene signals in FISH analysis after etoposide treatment (Figure 1C). The majority of the split FISH signals are most likely due chromosome translocations, rather than persistent DSBs. Since Ku, required for the tethering of DNA ends of DSBs, is functional in AT5BIVA and 11-4 cells, we speculate that most of the MLL gene rearrangements detected as split FISH signals are chromosome translocations [26]. Because DSBs associated with V(D)J recombination can persist in ATM deficient B cells [27], we could not completely exclude the possibility that a fraction of the rearrangement of the MLL gene detected as split MLL gene signals are persistent DSBs, Importantly, these V(D)J recombination-associated DSBs in ATM deficient B cells participate in chromosome translocations with other DSBs subsequently generated by ionizing irradiation [27]. Taken together, these findings further support the notion that ATM plays a role in the suppression of 11q23 chromosome translocations after etoposide treatment [12].

Classical nonhomologous DNA end-joining (NHEJ) has been shown to play a role in chromosome translocations [1], [28]. However, lymphomas with chromosome translocations are observed in classical NHEJ-defective, ligase IV/XRCC4 deficient, mice [29], [30]. Therefore, the involvement of alternative NHEJ processes, independent of ligase IV or XRCC4, has been also suggested in chromosome translocations [31], [32], [33], [34]. In the absence ATM kinase activity, aberrant metabolism of damaged DNA by recombinational repair proteins and chromatin remodelers might lead to alternative NHEJ processes resulting in 11q23 translocations after etoposide treatment. In replicating ATM deficient cells, these alternative NHEJ processes might co-opt activities of nucleases normally involved in replication and recombination, such as CtIP and MRE11 [35], that could remove the covalently bound topoisomerase II from the DNA break end before it can be patched up by the end-joining process. Since ATM deficiency increases the frequency of HR in mice and an increase in chromosomal translocations is observed in ATM-deficient cells [36], [37], [38], ATM may play a role in facilitating classical and repressing alternative NHEJ and/or HR pathways through the regulation of recombinational repair proteins and chromatin remodelers.

Our observation that etoposide induces the transient binding of RPA to the BCR suggests that this region is a specific target of etoposide (Figure 4A and B) and that the etoposide-induced DNA damage is processed into single-stranded DNA. RPA-single-stranded DNA complexes are common intermediates in checkpoint responses and DNA repair reactions [39]. The transient etoposide-induced RPA binding to the BCR occurs in ATM-proficient cells but in the absence of ATM it becomes more prolonged and spreads out into adjacent regions of the BCR (Figure 4A and B). This suggests that ATM is required to properly enable timely DNA repair through the regulation of the RPA binding. Recently, phosphorylation of RPA has been shown to impede HR by repressing the loading of RAD51 in PP4 phosphatase deficient cells [40]. Therefore, increased binding of hypophosphorylated RPA to the BCR after etoposide treatment may lead to the aberrant RAD51 loading in ATM deficient cells. We also show that the absence of ATM results in the excessive and prolonged binding of INO80 to the bt56/t56 regions (Figure 5C). Since INO80 exchanges RPA with RAD51 during HR, INO80, together with RPA, could be responsible for the aberrant loading of RAD51 [22].

We have identified the BCR in the MLL gene as the first native human chromosomal DNA locus where recombination proteins accumulate after induction of DNA damage by a specific DNA damaging agent. Although the nature of DNA damage induced by etoposide is still unclear, the BCR-specific DNA damage by etoposide, such as replication fork collapse could be involved in chromosome translocations. There were, however, no significant differences in the BrdU incorporation after etoposide treatment between the BCR and control beta-globin gene locus, suggesting that the incidence of replication fork stalling could be similar in the BCR and control locus (Figure S14). Therefore, ATM suppresses the prolonged accumulation of recombination proteins and thus our study suggests a mechanism through which the increased frequency of chromosome translocations observed in cells from AT patients occurs. DNA repair systems, especially HR, have emerged as novel therapeutic targets in anti-cancer therapy [41]. Our study implies that inhibiting HR can have additional benefits for an unexpected reason; the prevention of secondary malignancies triggered by anti-cancer therapies.

## Materials and Methods

### Cell culture and chemical treatment

BV173, a human B cell line derived from CML, was cultured in RPMI 1640 supplemented with 5% FBS. The SV40-transformed AT fibroblast cell line AT5BIVA and its ATM-proficient derivative, AT5BIVA cells reconstituted with chromosome 11 (11-4) [42], were kindly supplied by Dr. S. Matsuura. AT5BIVA cells were cultured in DMEM supplemented with 10% FBS. 11-4 cells were maintained in DMEM supplemented with 10% FBS and 0.2 mg/ml of G418. Cells were exposed to etoposide (Sigma, St Louis, MO) at 100 µM for 10 min or bleomycin (WAKO) at 30 µg/ml for 60 min, respectively. Dimethylsulfoxide (DMSO) was used as the vehicle for etoposide and was present in cell cultures at the final concentration of 0.1%. Bleomycin was dissolved in water. To inhibit ATM kinase activity, 11-4 cells were treated with 10 µM of ATM kinase specific inhibitor KU55933 for 2 hours before etoposide treatment.

### FISH analysis

FISH analysis was performed using LSI MLL Dual Color, BreakApart Rearrangement Probe (Vysis, Abbott Molecular Inc, IL, USA) according to the manufacturer's protocol. Images were acquired on a Zeiss AxioplanII microscope using an AxioCamMRm controlled by Axiovision. Subsequently, the cells containing split signals of abnormalities in at least 200 of etoposide-exposed or DMSO-exposed cells were counted. FISH analyses were repeated three times.

### Transient transfection of exogenous RAD51

Twenty-four hours before etoposide treatment AT5BIVA and 11-4 cells were transfected with the RAD51 expression vector pcDNA3.1RAD51 using GeneJuice transfection reagent (Novagen). The amount of expressed RAD51 was determined by analyzing immunoblots using ImageJ. The transfection efficiency was verified by calculating the percentage of flag-tag pcDNA3.1RAD51 expressed cells after immunofluorescence staining.

### Antibodies

The antibodies used for chromatin immunoprecipitation, immunoblotting and immunofluorescence staining were anti-RAD51 (Calbiochem), anti-RPA34 (LabVision), anti-glyceraldehyde-3-phosphate dehydrogenase (anti-GAPDH) (Santa Cruz), anti-beta-actin (Sigma), anti-Flag M2 (Sigma), anti-RAD21 (Abcam), and anti-INO80 [43] antibodies.

### Immunofluorescence staining

After fixation in 4% paraformaldehyde in 1x phosphate-buffered saline (PBS) for 10 min at room temperature, cells were permeabilized with 0.1% sodiumdodecyl sulfate (SDS)-0.5% Triton X-100 in 1x PBS for 5 min [19]. For the detection of RAD51 or Flag-tagged RAD51, fixed cells were incubated for 30 min at 37°C with anti-RAD51 antibody (1∶1,000) or anti-Flag M2 antibody (1∶200) in 1% bovine serum albumin/1xPBS. Fluorescein isothiocyanate (FITC)-conjugated goat anti-rabbit (1∶1,000, Biosource) antibodies was used as a secondary antibody. Nuclei were stained with Hoechst 33342. Cells were mounted using Vectashield and observed on an Axioplan2 microscope with AxioCamMRm controlled by Axiovision software (Zeiss).

### Primers

Primers for real-time PCR reactions were:

t2 forward: 5′CCACCTTTACAATGAGGAAGGA3′

t2 reverse: 5′CCCGACGTGGATTTTCTTTA3′,

t4 forward: 5′TTTGAGAACAAGTTGCAGACA3′

t4 reverse: 5′GGGACAATTGGTCAAACCTA3′

bbt56 forward: 5′TGGAAAGGACAAACCAGACC3′

bbt56 reverse: 5′AGTATTGGACATTGCGGGAG3′

bt56 forward: 5′TACTCTGAATCTCCCGCA3′

bt56 reverse: 5′CGCTCGTTCTCCTCTAA3′

t56 forward: 5′TTGCCAAGTCTGTTGTGAGC3′

t56 reverse: 5′CAGAGGCCCAGCTGTAGTTC3′

in14 forward: 5′GGTTCCCAACATATGGCTTT3′

in14 reverse: 5′GCCGCTCAGTACAGTTCACA3′

βG forward: 5′TTGGACCCAGAGGTTCTTTG3′

βG reverse: 5′GAGCCAGGCCATCACTAAAG3′

### Chromatin immunoprecipitation and real-time PCR assay

Chromatin immunoprecipitation was performed as described previously [44]. In brief, cells were fixed by adding formaldehyde to 1% final concentration for 10 min at room temperature. Cells were then sonicated to prepare chromatin suspensions of roughly 300–500 bps DNA in length. Immunoprecipitations were carried out using antibodies described in Methods. Normal rabbit/mouse IgG was used as a negative control. Real-time PCR reactions were carried out using LightCycle (Fast Start DNA Master SYBR GREEN 1, Roche or SYBR premix Ex Taq, TAKARA). Primers for real-time PCR were as described above. Dissociation curve analysis of the amplified DNA melting temperature showed that each primer set gave a single and specific product. The immunoprecipitation data were normalized to that of a control region in the β-globin gene (βG) to correct for experimental variation. The relative immunoprecipitation value represents the ratio of immunoprecipitated DNA after chemical treatment to immunoprecipitated DNA after vehicle treatment. All ChIP analyses were repeated at least three times and, in each experiment, quantitative PCR reactions were done in duplicate. Values represent the mean ± SE.

### Immunoblotting analyses

Cells were treated with etoposide, bleomycin or left untreated, unless stated otherwise. For immunoblotting analysis, samples were separated by SDS-PAGE, transferred to a PVDF membrane, and detected with antibodies against RAD51, RPA, INO80 and loading control proteins.

### Annexin V apoptosis assay

AT5BIVA and 11-4 cells were treated with vehicle (Con) or etoposide and allowed to recover in normal medium for 30 min (w30) and 6 hours (w6h). The Annexin V Apoptosis Assay was performed using Annexin V : PE Apoptosis Detection Kit according to the manufacturer's protocol and analyzed by flow cytometry (BD). Cells undergoing apoptosis were quantified as a percentage of total cells.

### Chromatin fraction preparation

Cells were collected, washed with phosphate-buffered saline (PBS) and resuspended in hypotonic buffer (10 mM Tris-HCl (pH 7.3), 10 mM KCl, 3 mM MgCl_2_). Briefly, cells were homogenized and vortexed before centrifuged for 5 min at 2, 900 rpm. The nuclear pellet was subsequently lysed with Low buffer (20 mM Tris-HCl (pH 7.3), 1.5 mM MgCl_2,_ 0.02 M KCl, 25% glycerol) and High buffer (20 mM Tris-HCl (pH 7.3), 1.5 mM MgCl_2,_ 1.2 M KCl, 25% glycerol). After rotation 60 min at 4°C, centrifuged for 14,000 rpm30 min. The insoluble chromatin pellet was sonicated in TGME buffer (50 mM Tris-HCl (pH 8.0), 5 mM MgCl_2_, 0.05 mM KCl, 25% glycerol).

### Long-distance inverse PCR (LDI-PCR)

Genomic DNA isolated from BIVA and 11-4# cells was digested with restriction enzyme *BamH*I (TOYOBO). Digested genomic DNA (2 µg) were self-ligated at 16°C for 24 hours using T4 DNA ligase (NEB). Subsequently, LDI-PCR reactions were performed using TaKaRa LA taq DNA polymerase (TAKARA). First round PCR conditions were 93°C 3 min; 10 cycles at 93°C 15sec, 60°C 30sec, and 68°C 8 min; 15 cycles at 93°C 15sec, 60°C 30sec. and 68°C 8 min (+20sec/cycle), 68°C 10 min. Second round nested PCR conditions were 93°C 3 min; 10 cycles at 93°C 15sec, 68°C 8 min; 20 cycles at 93°C 15sec, 60°C 30sec. and 68°C 8 min (+20sec/cycle); 68°C 10 min. PCR products were separated on 0.8% agarose gels. Non-germ-line DNA amplimers were gel extracted and sequenced directly. Primers for PCR were, (5′-3′)

1, GACATTCCCTTCTTCACTCTTTTCCTC

2, GCAGCCTCCACCACCAGAATCAGGTGAGTG

3, FTTTCGTGGAGGAGGCTCACTACTG

4, CAGCCTGGGTGACAAAGCAAAACAC

5, CCCATTAGCAGGTGGGTTTA

6, GAGGATCACGAGCCCACAAGGTCTA

7, AACCCTGCCCACTTGCCATTTGAAG

8, TTTGAGAACAAGTTGCAGACA

9, CCCACCCCACTCCTTTATATTCCCATAG

### Eletrophoretic mobility shift assay

Non-isotopic electrophoresis mobility shift assay was performed using nuclear extracts of AT5BIVA and 11-4 cells. The 56-bp dsDNA (5′-GATCAGTGATGGAGTTGGCCACTCCCTCTCTGCGCGCTCGCTCGCTCACTGAGGCC-3′) was end labeled with using DIG-11-ddUTP (Roche) [45]. DNA end-binding reaction and detection were performed according to the manufacturer's protocol (Roche). The DNA end-binding reactions were separated on 5% polyacrylamide gels. For the supershifts, the specific antibodies to Ku80 (Cell signaling) were added to the reaction mixture and incubated for 30 minutes before separating the DNA-protein complexes. Unlabeled dsDNA was used as specific competitor.

### Quantitative analysis of BrdU incorporation

Etoposide treated AT5BIVA and 11-4 cells were wash out and culture in 10 µM of BrdU containing or BrdU free medium for 60 min. Cells were sonicated to prepare chromatin suspensions of roughly 300–500 bps DNA in length. Chromatin fraction was treated by proteinase K and extracted with phenol/chloroform. Immunoprecipitations were carried out using antibodies against normal IgG or BrdU (Roche) after genomic DNA was denatured in boiling water for 10 min. Quantitative analysis of immunoprecipitated DNA was performed as described above**.**

## Supporting Information

Figure S1 Scheme of fluorescent probes for the 11q23 locus specific identifier (LSI). The maximal size of the overlap of the two contigs between exons 7 and 13 is 7.4 kb. Centromeric to the MLL breakpoint region is the 350-400 kb green probe and mostly telomeric to the BCR region is the 190 kb orange probe.
(0.15 MB TIF)

Figure S2 Dual-color FISH analysis of AT5BIVA and 11-4 cells. Cells were cultured for 36 hours (w36h) and 48 hours (w48h) in normal medium after etoposide treatment (Con; unexposed cells). The split signals (separated >1 µm) were counted from 200 cells. The experiments were performed three times (*P<0.001 as determined by the Z test of homogeneity for independent samples).
(0.17 MB TIF)

Figure S3 Analysis of apoptotic cells after etoposide treatment. AT5BIVA and 11-4 cells were treated with vehicle (Con) or etoposide and allowed to recover in normal medium for 30 min (w30) and 6 hours (w6h). Cells were incubated with Annexin V-PE in a buffer containing 7-Amino-actinomycin (7-AAD) and analyzed by flow cytometry. Cells undergoing apoptosis were quantified as a percentage of total cells.
(0.38 MB TIF)

Figure S4 The expression level and DNA binding activity of Ku80. (A) Immunoblotting analysis of Ku80 using anti-Ku80 antibodies. (B) DNA end-binding activity of Ku70/80. Non-isotopic electrophoretic mobility shift assay was performed using nuclear extracts of AT5BIVA and 11-4 cells. Lane 1: BIVA; Lane 2: BIVA + anti-Ku80; Lane 3: 11-4; Lane 4: 11-4 + anti-Ku80; Lane 5: unlabeled specific probe.
(0.81 MB TIF)

Figure S5 Summary of etoposide induced breakpoints within MLL BCR. (A) LDI (Long Distance Inverse) PCR was performed using BamHI digested genomic DNA. (B) The spectrum of etoposide-induced MLL gene repair products. Before isolating genomic DNA, AT5BIVA and 11-4 cells were treated with 100 µM of etoposide for 10 min and allowed to recover for 6 or 24 hours in normal medium. (C) The sequence of breakpoint junctions. Black: MLL gene; Blue: partner gene; Red: micro-homology sequence between MLL gene and fusion partner gene.
(0.71 MB TIF)

Figure S6 The expression of exogenous RAD51. 11-4 cells were transfected with pcDNA3.1flagRAD51expression plasmid for 24 hours. Immunofluorescence staining was performed with anti-Flag M2 antibodies. Blue: DNA, Red: flagRAD51. Scale bar: 20 µm.
(1.54 MB TIF)

Figure S7 Immunoblotting analysis of RAD51 overexpressing cells. Chromatin fraction (Chr.f) and whole cell extracts (WCE) were isolated from 100 µM of etoposide (VP), or vehicle (Con) treated cells. AT5BIVA and 11-4 cells were transfected with an empty expression vector (mock) or RAD51 expression vector (RAD51) for 24 hours before etoposide treatment.
(0.31 MB TIF)

Figure S8 Translocation breakpoint hotspot in the BCR. The chromosomal translocation breakpoint hotspot is shown in red. Boxes indicate the presumed topoisomerase II binding sites. Arrows indicate primer sets (t4, bbt56, bt56 and t56) used for real-time PCR.
(0.65 MB TIF)

Figure S9 Amplification efficiency of genomic DNA by PCR. Real-time PCR analysis of genomic DNA extracted from AT5BIVA, 11-4 and BV173 cells using the primers indicated in Figure 3A and the Methods. Values represent the means+/-SE from three independent experiments.
(0.27 MB TIF)

Figure S10 Binding of RAD51 to BCR in the MLL gene after a high dose of etoposide. ChIP analysis of AT5BIVA and 11-4 cells was performed with anti-RAD51 antibodies, immediately after 300 µM of etoposide (VP), or vehicle (Con) exposure, and 30 min after recovery in normal medium (w30). DNA was analyzed by real-time PCR using the primers indicated in Figure 3A and the Methods. Values represent the means+/-SE from three independent experiments. Asterisks represent P<0.05 (compared to β-globin by Student's t test).
(0.25 MB TIF)

Figure S11 Binding of RAD51 to breakpoint clustering region in the MLL gene after bleomycin treatment. (A) RAD51 expression after etoposide or bleomycin treatment. Immunoblotting analysis of BV173 cells using the anti-RAD51 antibody. BV173 cells were treated with etoposide at 100 (100) or 120 (120) µM for 10 min, or bleomycin at 30 µg/ml for 60 min (BLM), and allowed to recover in normal medium for 30 min (100w, 120w, 30w). GAPDH was used as a loading control. (B, C) ChIP analysis after bleomycin treatment of 11-4 (B) and AT5BIVA (C) cells using anti-RAD51 antibodies, immediately after bleomycin at 30 µg/ml for 60 min (BLM), or vehicle (Con) exposure, and 30 min after recovery in normal medium (w30).
(0.28 MB TIF)

Figure S12 Binding of RAD51 to BCR in the MLL gene after ATM inhibitor treatment. Following treatment of cells with 10 µM of ATM inhibitor (KU55933) for 2 hours, ChIP analysis of 11-4 cells was performed with anti-RAD51 antibodies, immediately after etoposide (VP), or vehicle (Con) exposure, and 30 min after recovery in normal medium (w30), DNA was analyzed by real-time PCR. Values represent the ratio of enrichment of RAD51 in ATM inhibitor treated cells to untreated cells.
(0.19 MB TIF)

Figure S13 Anti-RPA antibodies immunoprecipitate both phospho-RPA and an unmodified form. Immunoprecipitation analysis was performed using anti-RPA32 antibodies. Cell lysates were prepared from ATBIVA and 11-4 cells, immediately after etoposide (VP), or vehicle (Con) exposure, or 30 min (w30) and 2 hours (w2h) after recovery in normal medium. Immunoprecipitates and input (1% of cell lysates) were separated by SDS-PAGE and blotted with anti-RPA32 antibodies. IP: immunoprecipitation. WB: western blotting. RPA32-P: phospho-RPA.
(0.18 MB TIF)

Figure S14 BrdU incorporation after etoposide treatment. (A) Immunofluorescence staining were performed using cells with or without etoposide exposure, followed by BrdU labeling for 60 min. Scale bar: 20 µm. (B) Quantitative analysis of immunoprecipitated DNA using real-time PCR. Genomic DNA was isolated from cells with or without etoposide exposure, followed by BrdU labeling for 60 min. 15 µg of sonicated and purified DNA was immunoprecipitated by normal IgG or anti-BrdU antibodies.
(1.58 MB TIF)
