# Supplementary figures and images for: ATM Modulates the Loading of Recombination Proteins onto a Chromosomal Translocation Breakpoint Hotspot
Source: PLoS One. 2010 Oct 27;5(10):e13554. doi: 10.1371/journal.pone.0013554 (PMC2965082; doi:10.1371/journal.pone.0013554)

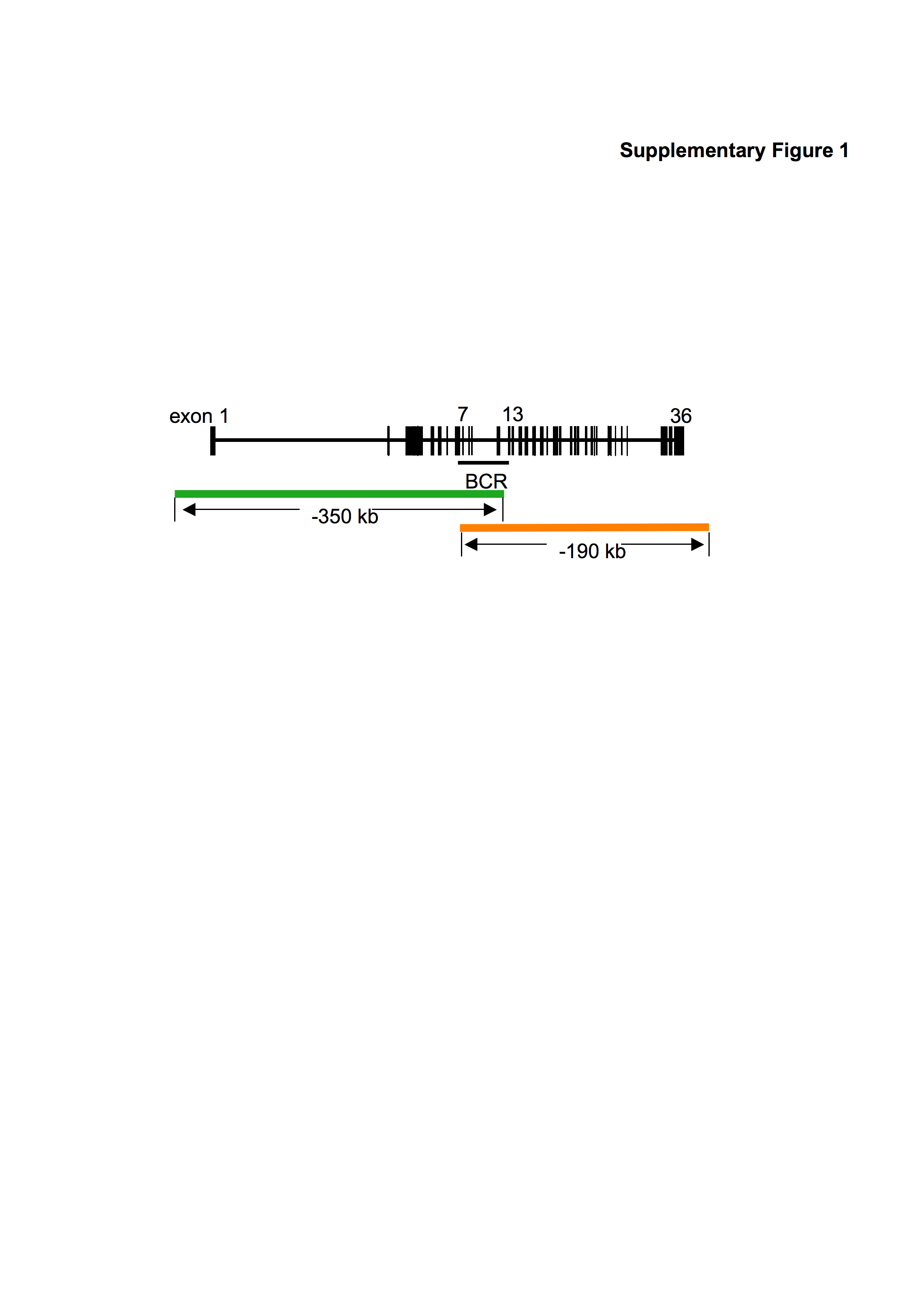

Supplement: Figure S1 — Scheme of fluorescent probes for the 11q23 locus specific identifier (LSI). The maximal size of the overlap of the two contigs between exons 7 and 13 is 7.4 kb. Centromeric to the MLL breakpoint region is the 350-400 kb green probe and mostly telomeric to the BCR region is the 190 kb orange probe. (0.15 MB TIF) [file pone.0013554.s001.tif]

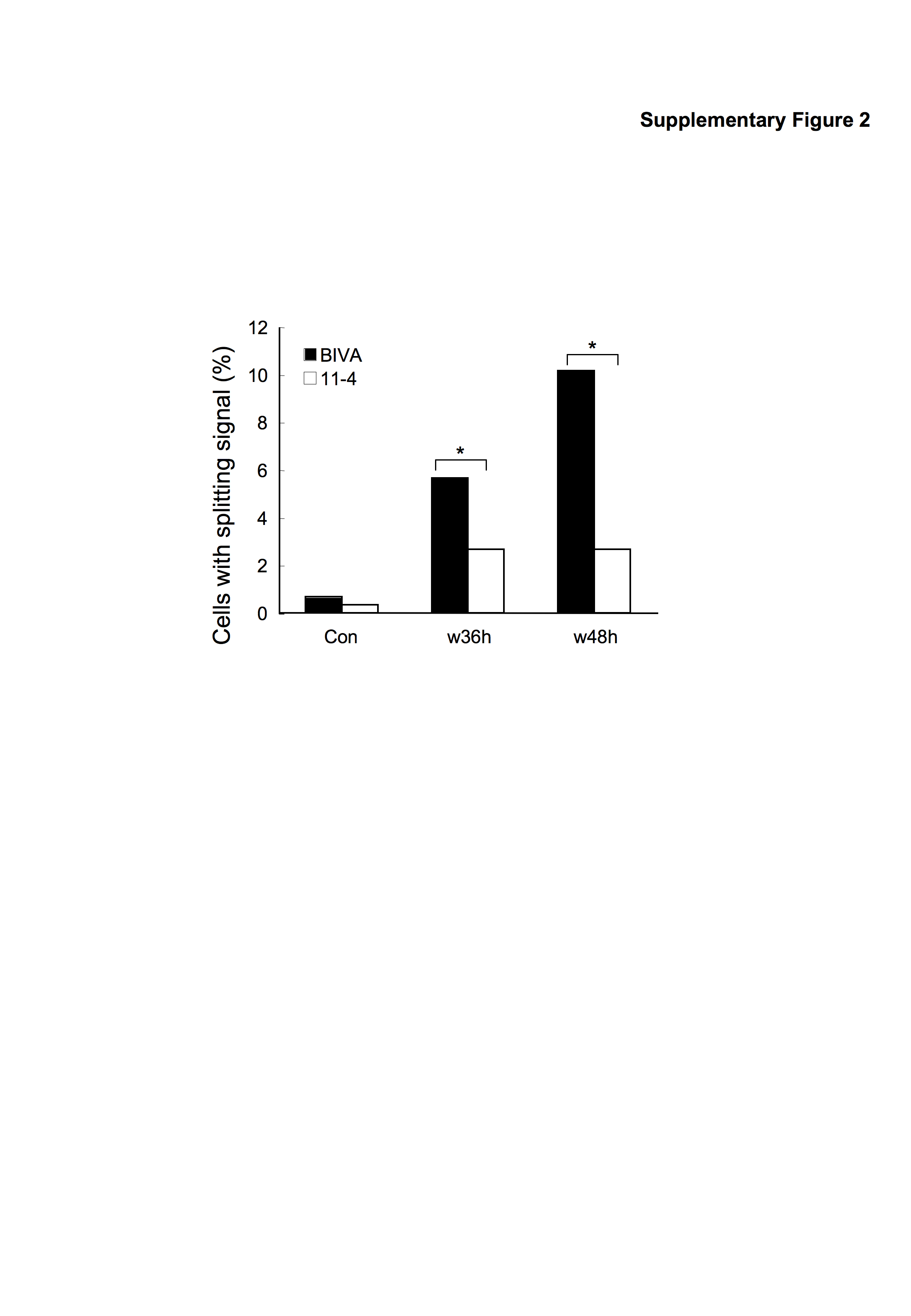

Supplement: Figure S2 — Dual-color FISH analysis of AT5BIVA and 11-4 cells. Cells were cultured for 36 hours (w36h) and 48 hours (w48h) in normal medium after etoposide treatment (Con; unexposed cells). The split signals (separated >1 µm) were counted from 200 cells. The experiments were performed three times (*P<0.001 as determined by the Z test of homogeneity for independent samples). (0.17 MB TIF) [file pone.0013554.s002.tif]

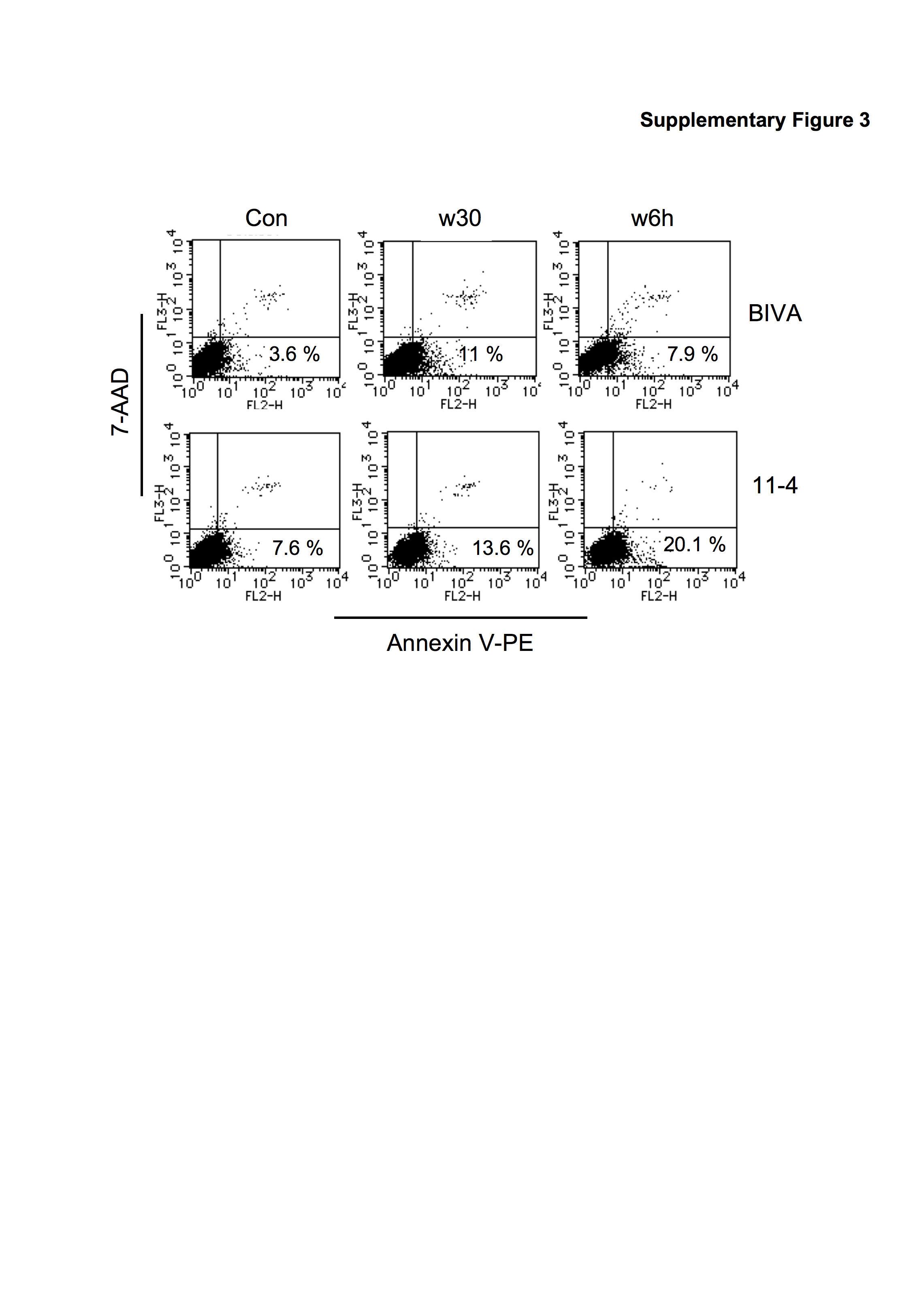

Supplement: Figure S3 — Analysis of apoptotic cells after etoposide treatment. AT5BIVA and 11-4 cells were treated with vehicle (Con) or etoposide and allowed to recover in normal medium for 30 min (w30) and 6 hours (w6h). Cells were incubated with Annexin V-PE in a buffer containing 7-Amino-actinomycin (7-AAD) and analyzed by flow cytometry. Cells undergoing apoptosis were quantified as a percentage of total cells. (0.38 MB TIF) [file pone.0013554.s003.tif]

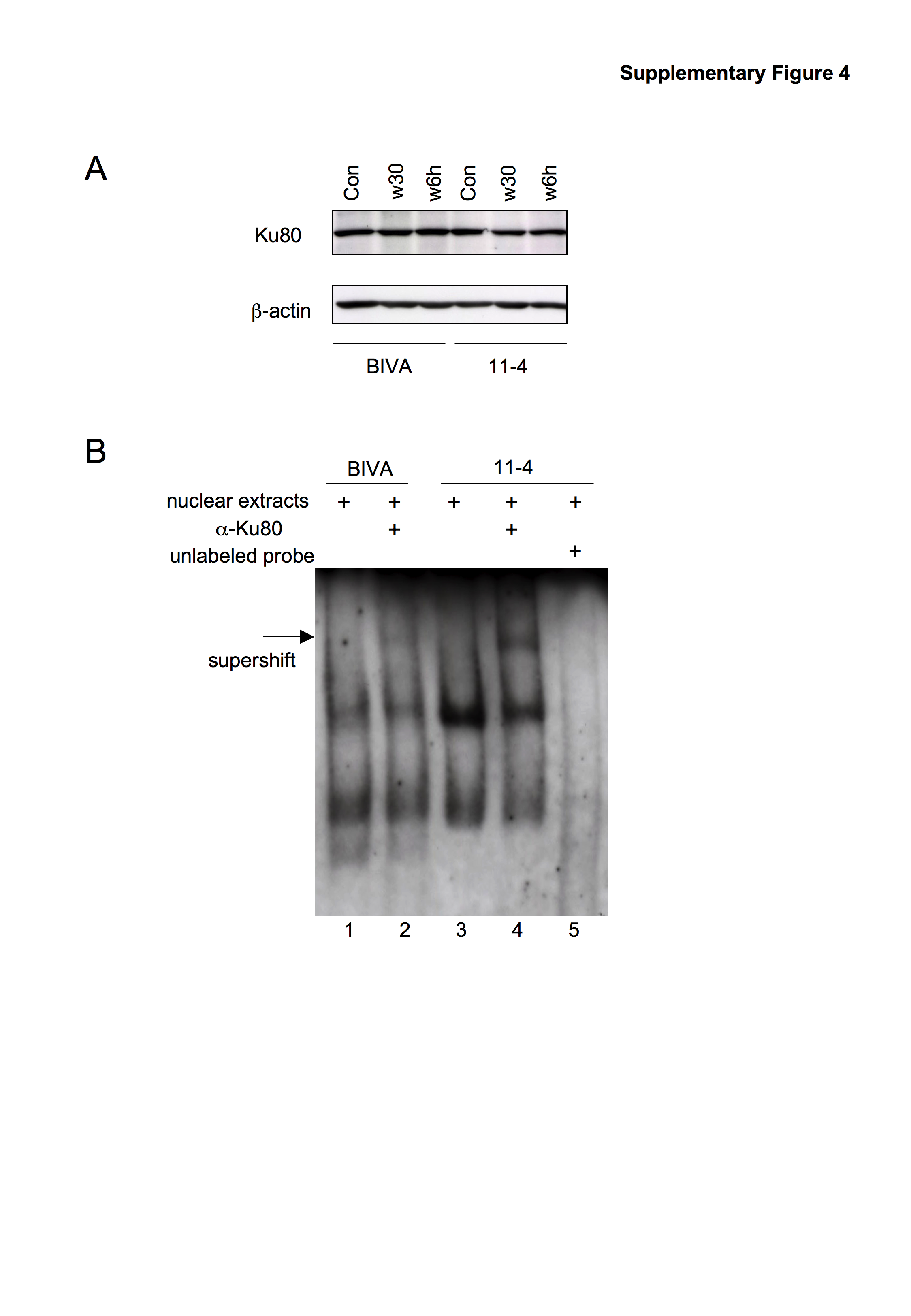

Supplement: Figure S4 — The expression level and DNA binding activity of Ku80. (A) Immunoblotting analysis of Ku80 using anti-Ku80 antibodies. (B) DNA end-binding activity of Ku70/80. Non-isotopic electrophoretic mobility shift assay was performed using nuclear extracts of AT5BIVA and 11-4 cells. Lane 1: BIVA; Lane 2: BIVA + anti-Ku80; Lane 3: 11-4; Lane 4: 11-4 + anti-Ku80; Lane 5: unlabeled specific probe. (0.81 MB TIF) [file pone.0013554.s004.tif]

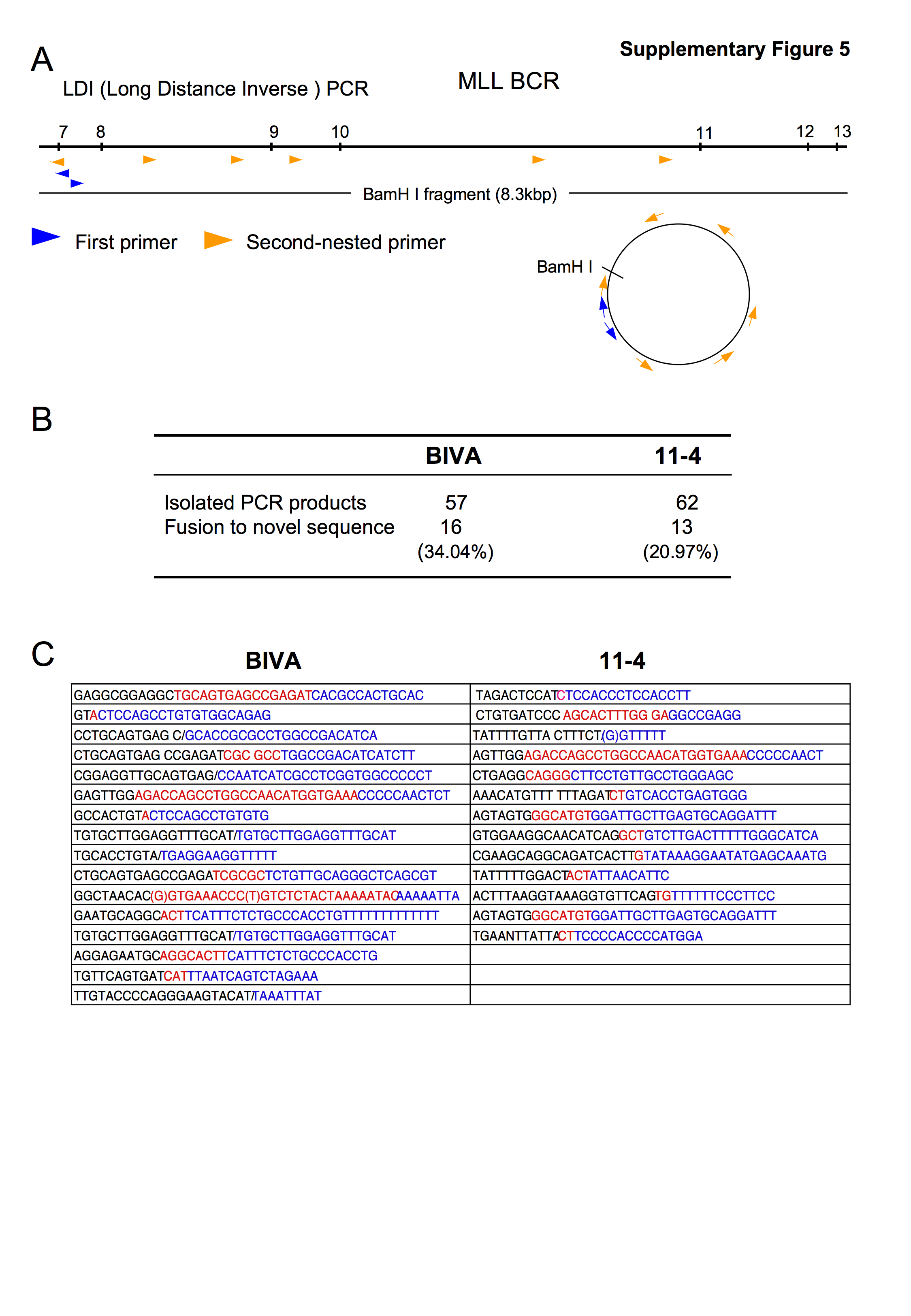

Supplement: Figure S5 — Summary of etoposide induced breakpoints within MLL BCR. (A) LDI (Long Distance Inverse) PCR was performed using BamHI digested genomic DNA. (B) The spectrum of etoposide-induced MLL gene repair products. Before isolating genomic DNA, AT5BIVA and 11-4 cells were treated with 100 µM of etoposide for 10 min and allowed to recover for 6 or 24 hours in normal medium. (C) The sequence of breakpoint junctions. Black: MLL gene; Blue: partner gene; Red: micro-homology sequence between MLL gene and fusion partner gene. (0.71 MB TIF) [file pone.0013554.s005.tif]

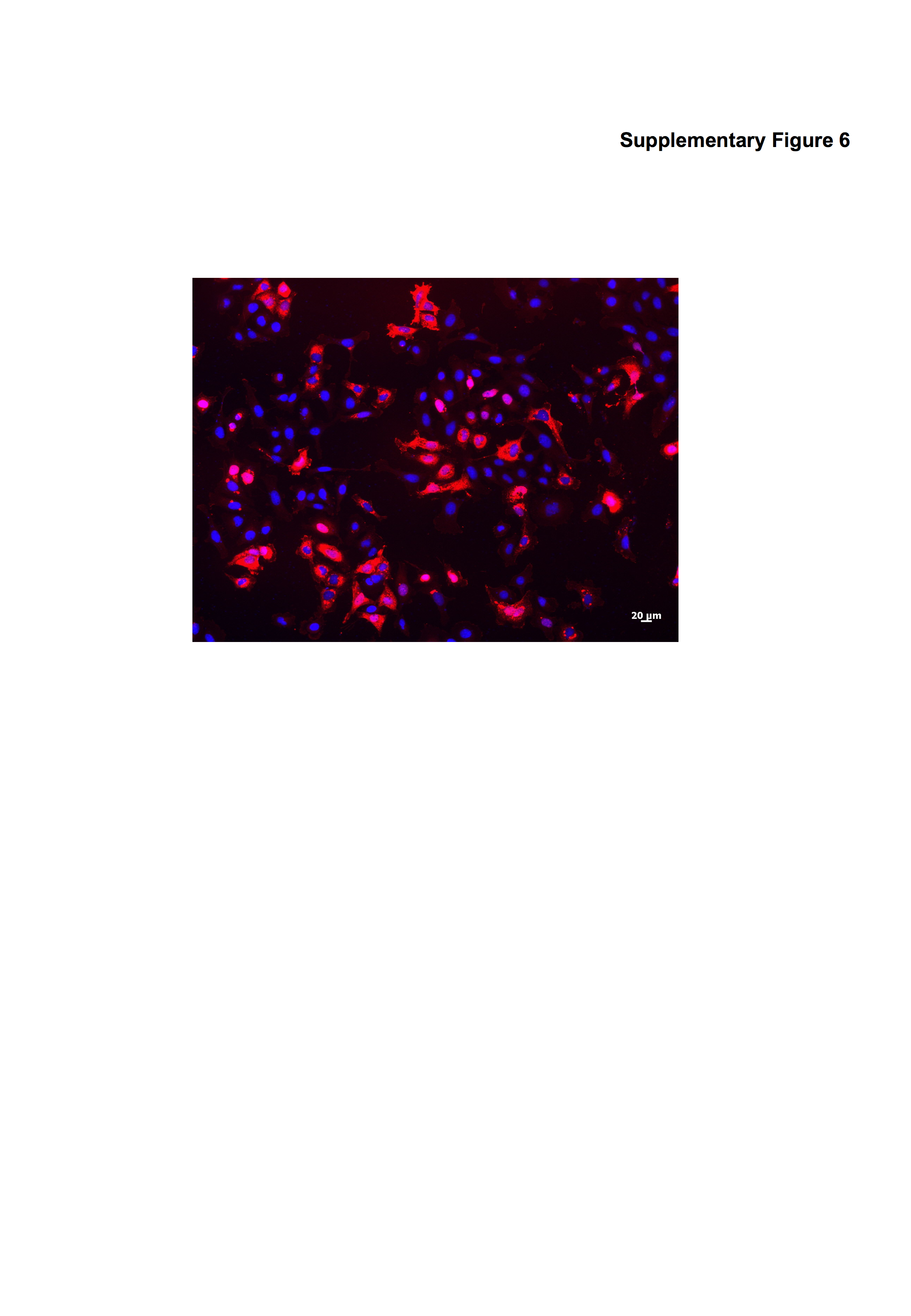

Supplement: Figure S6 — The expression of exogenous RAD51. 11-4 cells were transfected with pcDNA3.1flagRAD51expression plasmid for 24 hours. Immunofluorescence staining was performed with anti-Flag M2 antibodies. Blue: DNA, Red: flagRAD51. Scale bar: 20 µm. (1.54 MB TIF) [file pone.0013554.s006.tif]

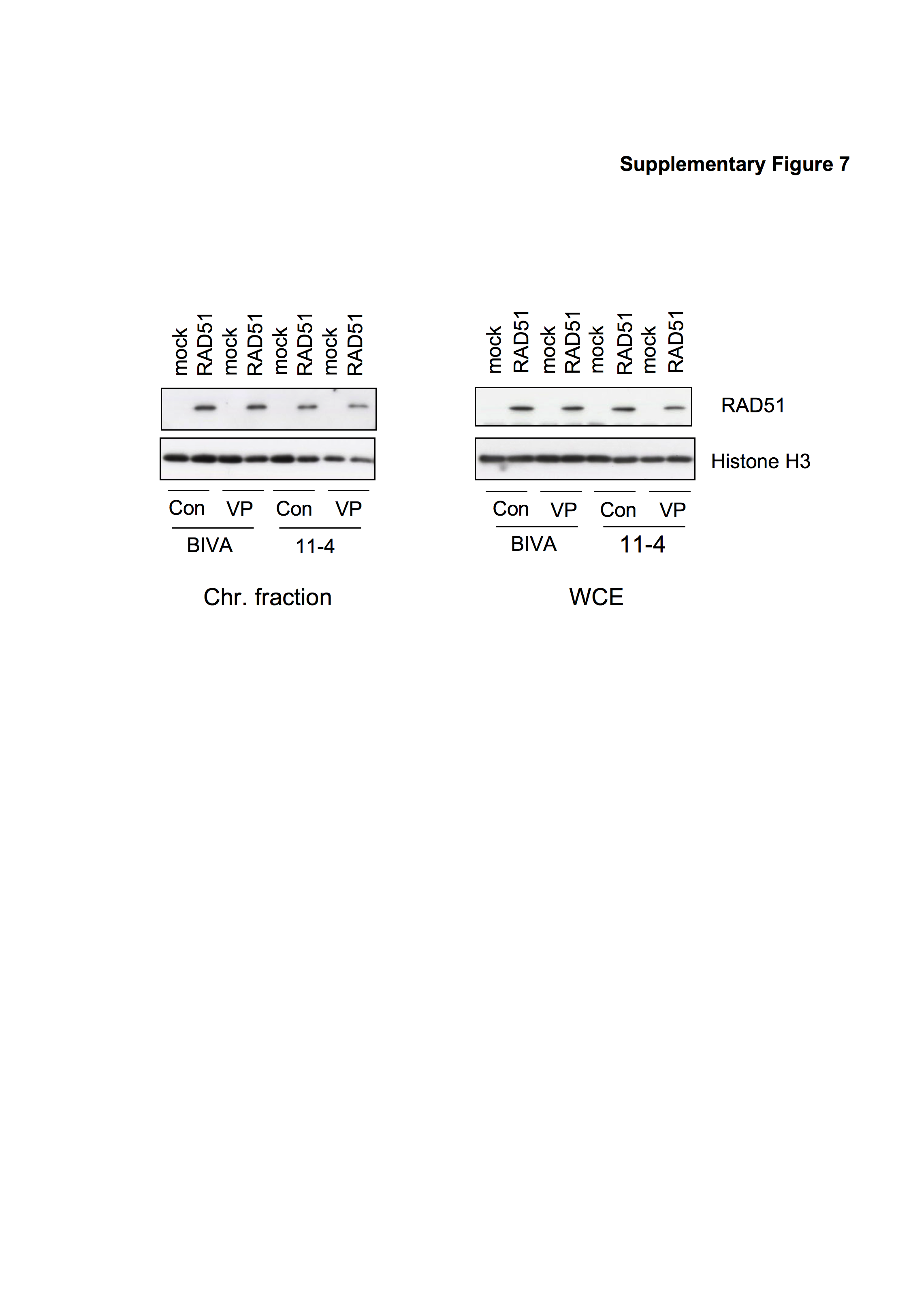

Supplement: Figure S7 — Immunoblotting analysis of RAD51 overexpressing cells. Chromatin fraction (Chr.f) and whole cell extracts (WCE) were isolated from 100 µM of etoposide (VP), or vehicle (Con) treated cells. AT5BIVA and 11-4 cells were transfected with an empty expression vector (mock) or RAD51 expression vector (RAD51) for 24 hours before etoposide treatment. (0.31 MB TIF) [file pone.0013554.s007.tif]

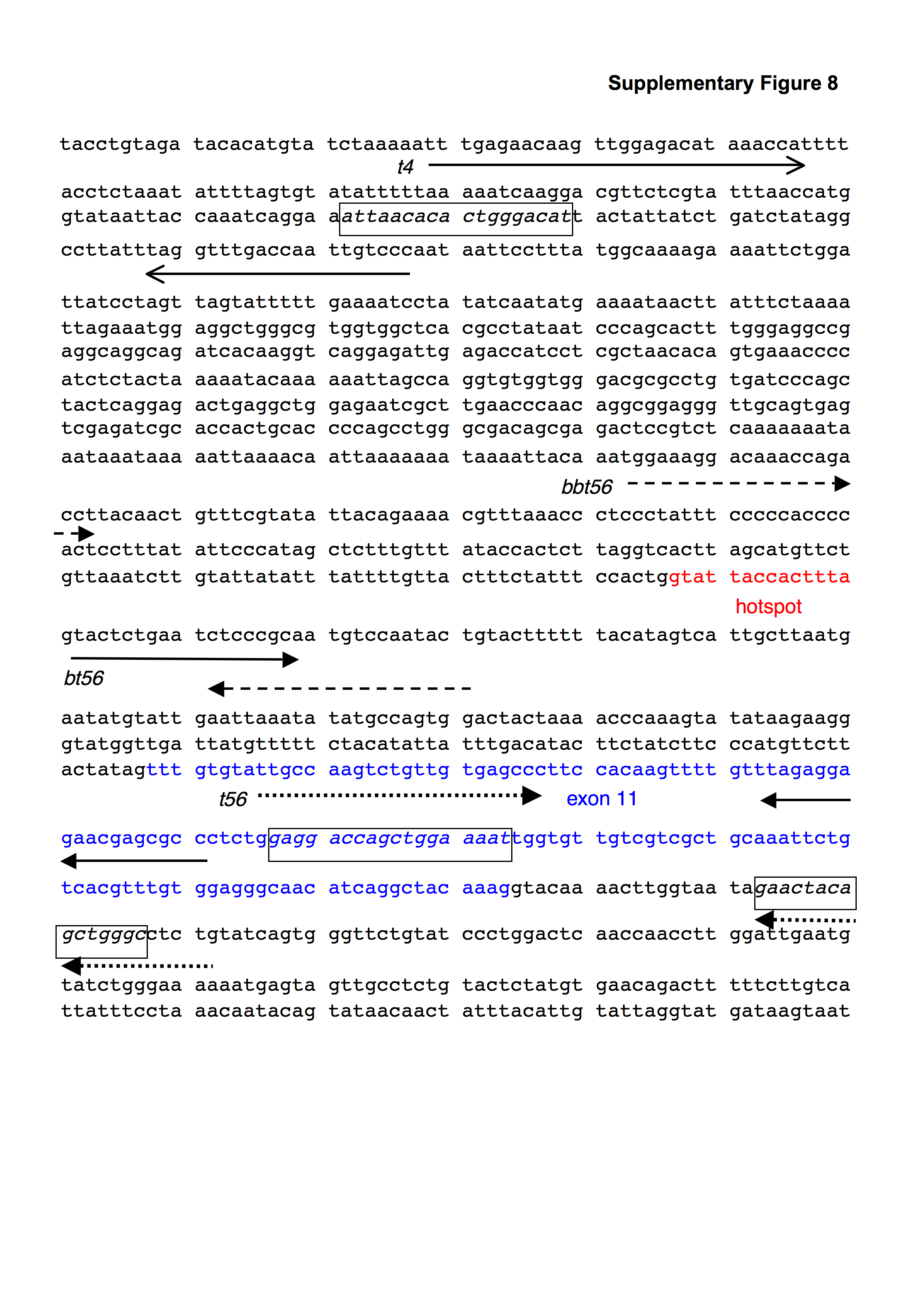

Supplement: Figure S8 — Translocation breakpoint hotspot in the BCR. The chromosomal translocation breakpoint hotspot is shown in red. Boxes indicate the presumed topoisomerase II binding sites. Arrows indicate primer sets (t4, bbt56, bt56 and t56) used for real-time PCR. (0.65 MB TIF) [file pone.0013554.s008.tif]

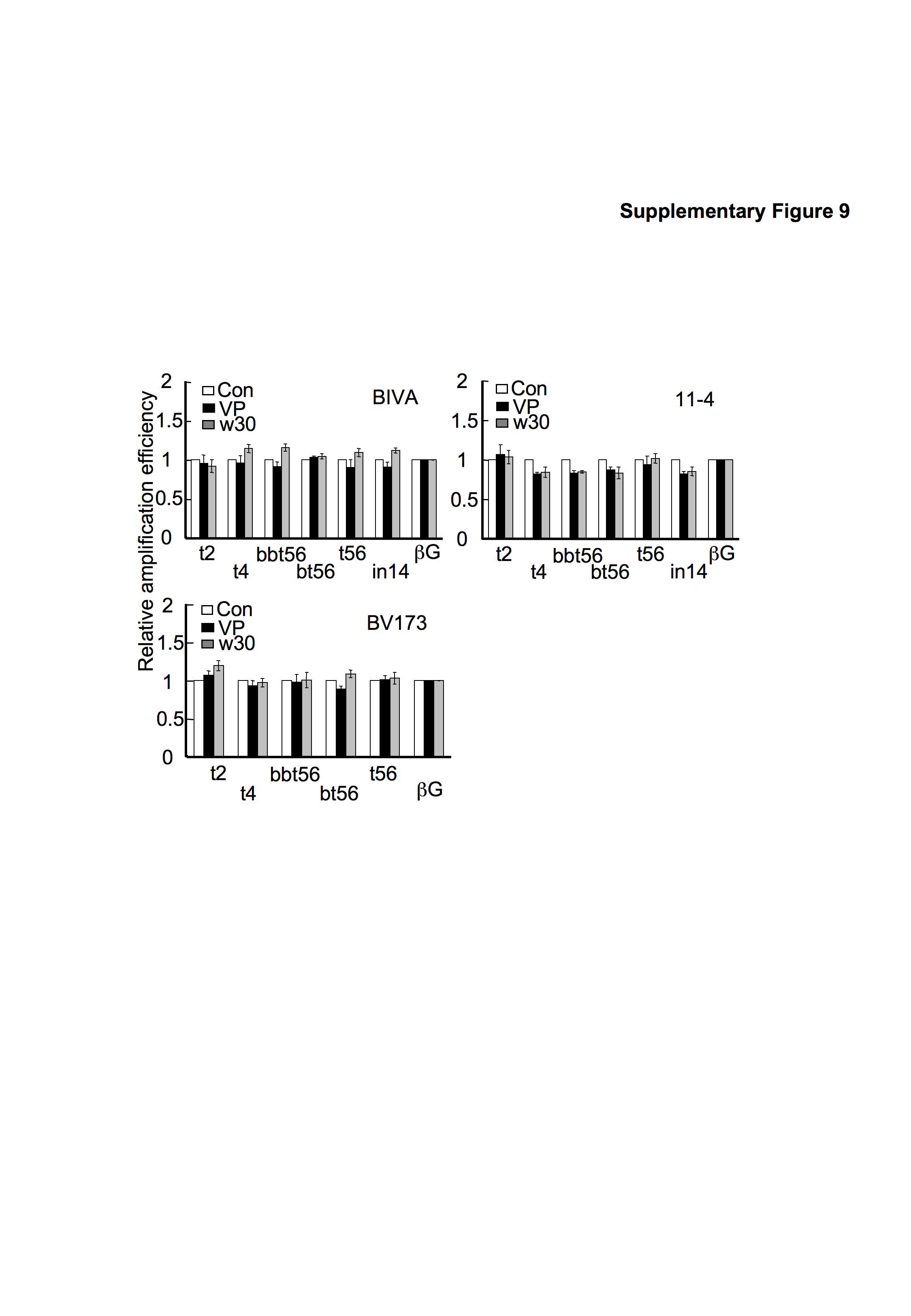

Supplement: Figure S9 — Amplification efficiency of genomic DNA by PCR. Real-time PCR analysis of genomic DNA extracted from AT5BIVA, 11-4 and BV173 cells using the primers indicated in Figure 3A and the Methods. Values represent the means+/-SE from three independent experiments. (0.27 MB TIF) [file pone.0013554.s009.tif]

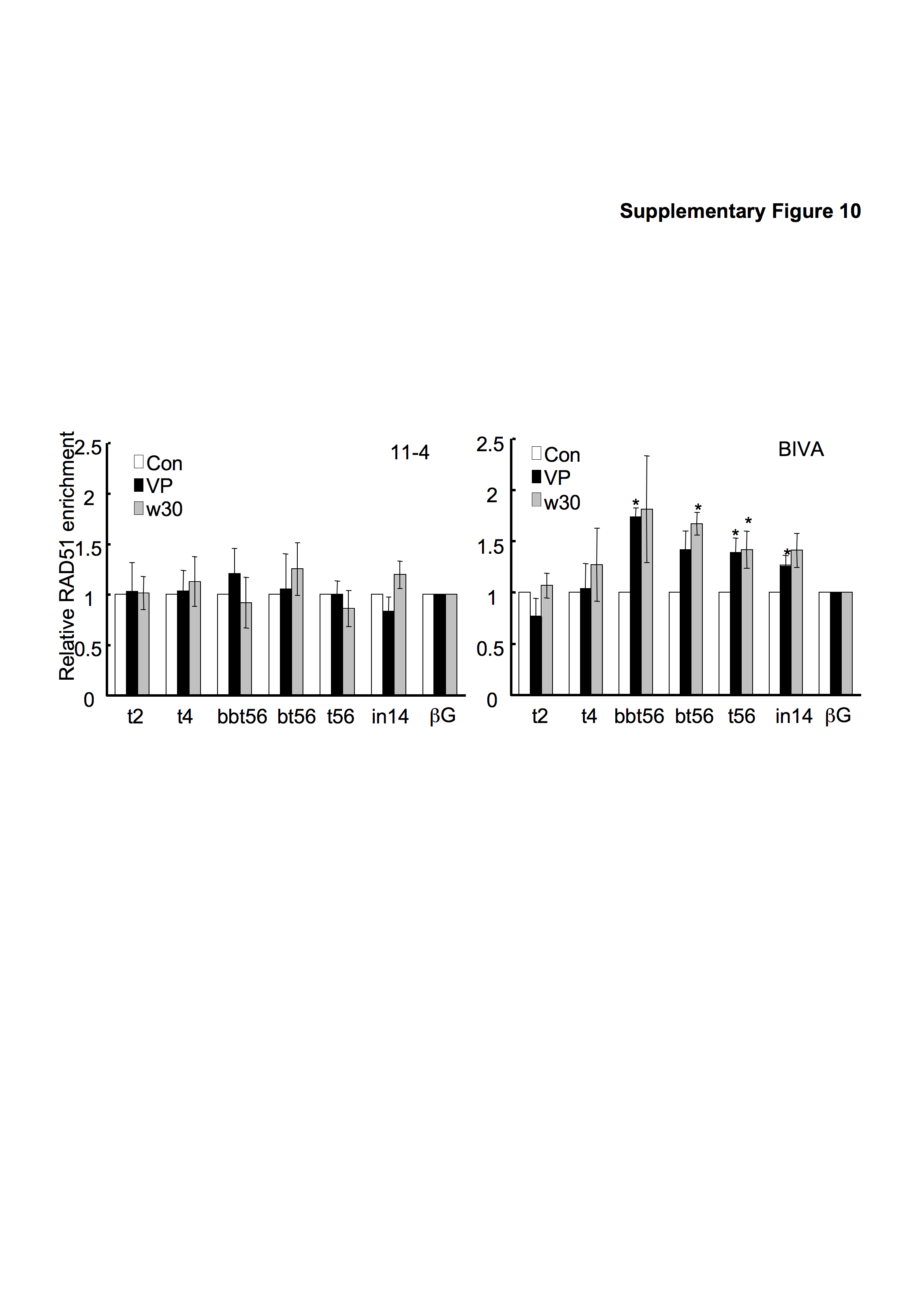

Supplement: Figure S10 — Binding of RAD51 to BCR in the MLL gene after a high dose of etoposide. ChIP analysis of AT5BIVA and 11-4 cells was performed with anti-RAD51 antibodies, immediately after 300 µM of etoposide (VP), or vehicle (Con) exposure, and 30 min after recovery in normal medium (w30). DNA was analyzed by real-time PCR using the primers indicated in Figure 3A and the Methods. Values represent the means+/-SE from three independent experiments. Asterisks represent P<0.05 (compared to β-globin by Student's t test). (0.25 MB TIF) [file pone.0013554.s010.tif]

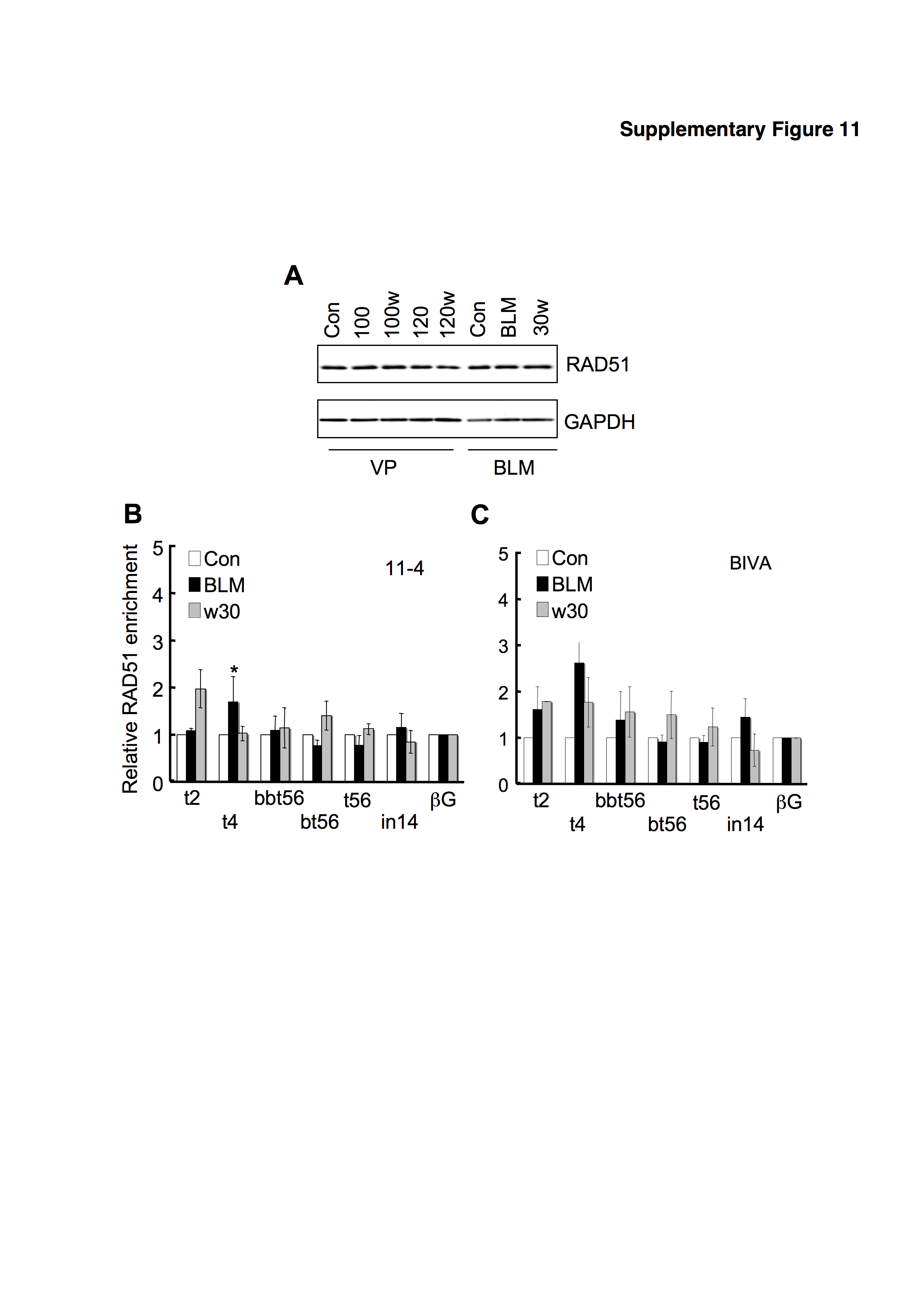

Supplement: Figure S11 — Binding of RAD51 to breakpoint clustering region in the MLL gene after bleomycin treatment. (A) RAD51 expression after etoposide or bleomycin treatment. Immunoblotting analysis of BV173 cells using the anti-RAD51 antibody. BV173 cells were treated with etoposide at 100 (100) or 120 (120) µM for 10 min, or bleomycin at 30 µg/ml for 60 min (BLM), and allowed to recover in normal medium for 30 min (100w, 120w, 30w). GAPDH was used as a loading control. (B, C) ChIP analysis after bleomycin treatment of 11-4 (B) and AT5BIVA (C) cells using anti-RAD51 antibodies, immediately after bleomycin at 30 µg/ml for 60 min (BLM), or vehicle (Con) exposure, and 30 min after recovery in normal medium (w30). (0.28 MB TIF) [file pone.0013554.s011.tif]

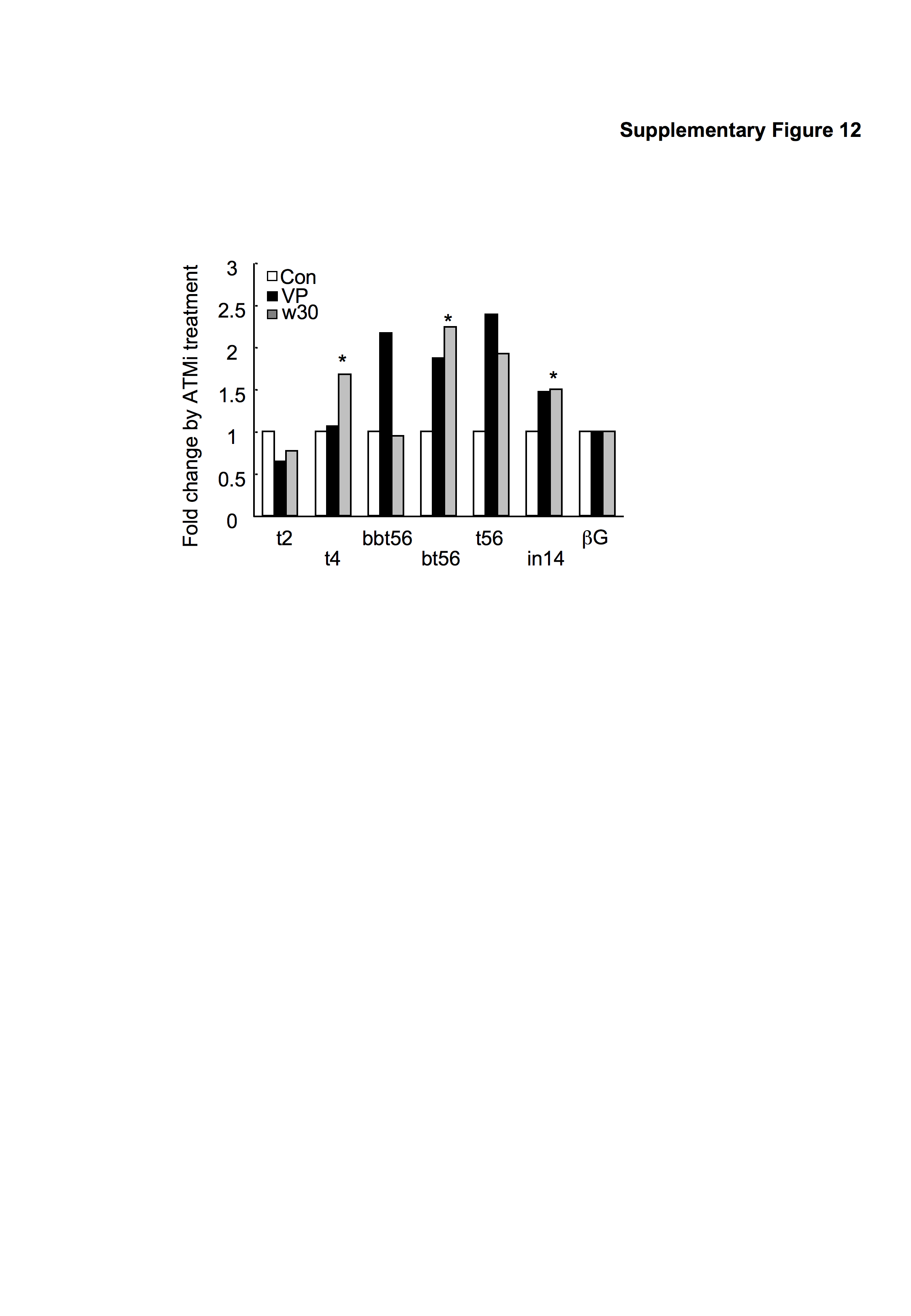

Supplement: Figure S12 — Binding of RAD51 to BCR in the MLL gene after ATM inhibitor treatment. Following treatment of cells with 10 µM of ATM inhibitor (KU55933) for 2 hours, ChIP analysis of 11-4 cells was performed with anti-RAD51 antibodies, immediately after etoposide (VP), or vehicle (Con) exposure, and 30 min after recovery in normal medium (w30), DNA was analyzed by real-time PCR. Values represent the ratio of enrichment of RAD51 in ATM inhibitor treated cells to untreated cells. (0.19 MB TIF) [file pone.0013554.s012.tif]

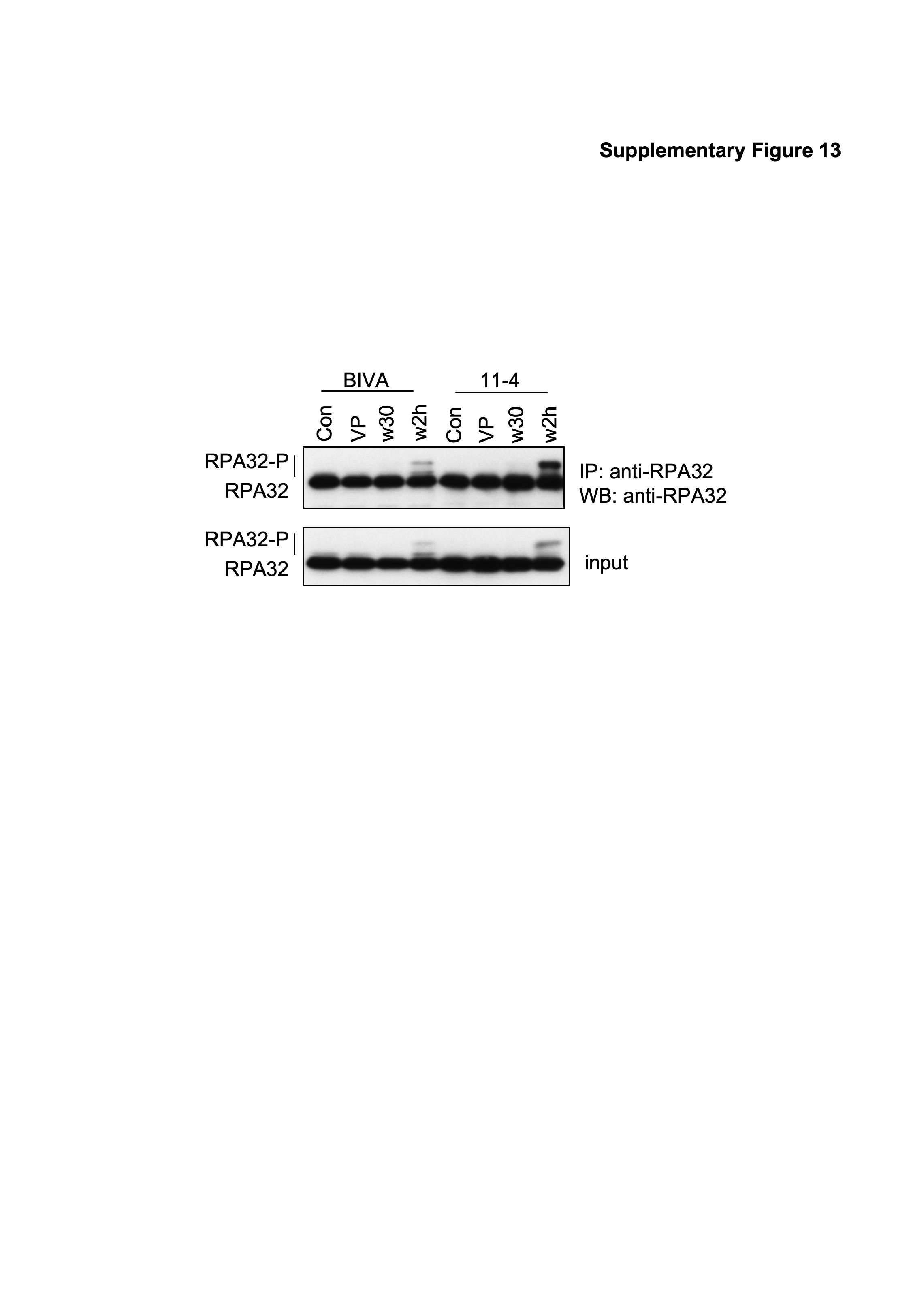

Supplement: Figure S13 — Anti-RPA antibodies immunoprecipitate both phospho-RPA and an unmodified form. Immunoprecipitation analysis was performed using anti-RPA32 antibodies. Cell lysates were prepared from ATBIVA and 11-4 cells, immediately after etoposide (VP), or vehicle (Con) exposure, or 30 min (w30) and 2 hours (w2h) after recovery in normal medium. Immunoprecipitates and input (1% of cell lysates) were separated by SDS-PAGE and blotted with anti-RPA32 antibodies. IP: immunoprecipitation. WB: western blotting. RPA32-P: phospho-RPA. (0.18 MB TIF) [file pone.0013554.s013.tif]

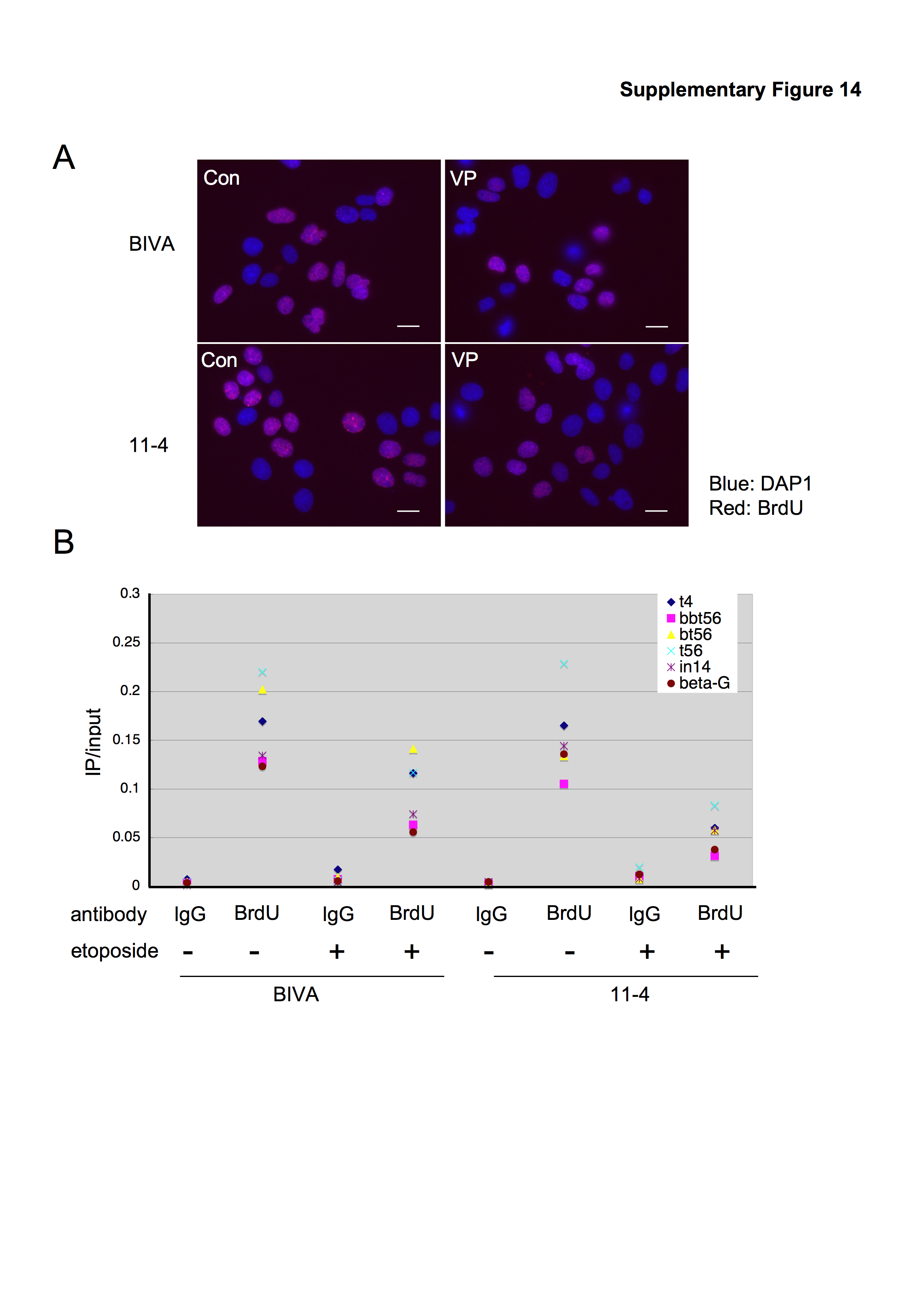

Supplement: Figure S14 — BrdU incorporation after etoposide treatment. (A) Immunofluorescence staining were performed using cells with or without etoposide exposure, followed by BrdU labeling for 60 min. Scale bar: 20 µm. (B) Quantitative analysis of immunoprecipitated DNA using real-time PCR. Genomic DNA was isolated from cells with or without etoposide exposure, followed by BrdU labeling for 60 min. 15 µg of sonicated and purified DNA was immunoprecipitated by normal IgG or anti-BrdU antibodies. (1.58 MB TIF) [file pone.0013554.s014.tif]
